# Blood Matrices and Sample Preparation Influence Blood Marker Discovery

**DOI:** 10.1021/acs.jproteome.5c00836

**Published:** 2025-12-16

**Authors:** Thomas F. Gronauer, Juliane Merl-Pham, Christine von Toerne, Katharina Habler, Daniel Teupser, Stefanie M. Hauck

**Affiliations:** † Metabolomics and Proteomics Core (MPC), Helmholtz Zentrum München, 9150 German Research Center for Environmental Health (GmbH) 80939 Munich, Germany; ‡ Institute of Laboratory Medicine, 27192 LMU University Hospital, LMU Munich, 81377 Munich, Germany

**Keywords:** plasma, serum, iST, ENRICH-iST, perCA, SPEED, Mag-Net, mpwR, proteomics, biomarkers

## Abstract

While plasma and serum are widely used in high-throughput proteomics, the impact of different blood matrix types remains underexplored. Routine diagnostics most commonly use serum or Li-heparin plasma, while the proteomics community primarily focuses on advancing analytical depth in EDTA plasma. Here, we systematically investigated the LC–MS/MS proteomic profiles of pooled blood samples from three healthy, voluntary probands including serum (with/without separation gel) and plasma anticoagulated with EDTA, citrate, or Li-heparin. Sample preparation was conducted with the commercially available iST and ENRICH-iST kits, strong-anion exchange (SAX) beads, the TFA-based approach SPEED, and perchloric acid (perCA) precipitation. Mass-spectrometric measurements were performed on a Q Exactive HF-X and a timsTOF HT in data-independent acquisition mode (DIA). Protein identifications varied considerably across matrix types with EDTA plasma and serum outperforming citrate plasma. Sample preparation methods with SAX beads, ENRICH-iST, and perCA yielded the highest identification numbers but also showed increased variability. Across all samples, 181 protein groups overlapped for timsTOF HT data. Subsets of protein groups were specific for the matrix and preparation. This study shows a systematic approach to determining suitable sample preparation and matrix parameters for the robust identification of individual body fluid marker proteins by mass spectrometry.

## Graphical abstract

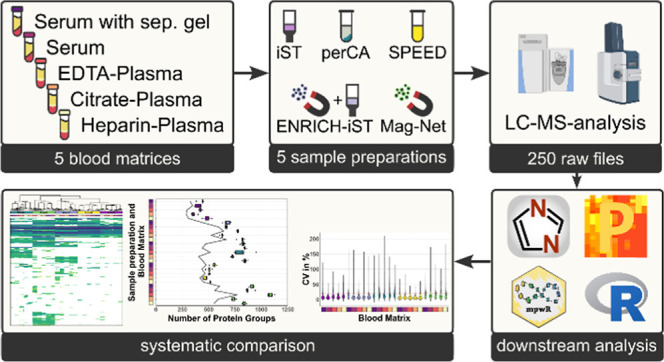

## Introduction

In medical research, blood is the primary body fluid that is investigated to decipher molecular processes in human diseases. While blood samples are easily accessible and available with minimally invasive procedures, mass spectrometry-based proteomics approaches for biomarker discovery in blood and other body fluids face various challenges. The high dynamic range of plasma proteins impairs the analytical depths and coverage.^1^ The susceptibility to minor changes during clinical sample collection and preparation such as variation in centrifugation steps,^2^ collection tubes,^3,4^ sample temperature,^5^ and handling critically affects sample composition. Various studies have elaborated on similarities and differences in proteomic profiles of blood matrices such as EDTA plasma, heparin plasma, and serum.^4–6^ Other studies showed influences of blood collection tube components on clinical assay outcomes.^3^ A recent study showed that commercially available plasma should also be treated with caution, as the protein content can vary greatly in some cases and there is usually no detailed information on the preparation.^7^ Therefore, over the years, guidelines have been proposed to ensure the appropriate usage, handling, and storage of these valuable research samples.^8–11^

Today, most of the assays in clinical laboratory medicine are based on serum or Li-heparinized plasma. At the same time, the proteomics community has composed guidelines that should ensure reproducibility and validity in LC–MS-based proteomic biomarker discovery.^12^ These recommendations primarily comprise the use of EDTA-anticoagulated blood,^8,11^ since other coagulants may hamper protein identification and quantification.^11^ This complicates the comparability of clinical assay outcomes with LC–MS-based biomarker identification and might additionally slow the implementation of mass spectrometry in the daily clinical routine.

In a joint effort, we recently proved that, in principle, the accurate and reproducible identification and quantification of 22 FDA-approved biomarkers over four months and three independent time points is possible with different LC–MS setups and laboratory settings.^13^ However, it is still difficult to substantially increase the number of proteins identified from neat plasma without long fractionation steps.

Another crucial parameter for the success of protein identification and quantification from different blood matrices is the applied sample preparation workflow. The high dynamic range of plasma proteins in unmodified neat plasma or serum notoriously leads to low protein IDs—a disadvantage that can only be offset to a limited extent using advanced LC–MS setups.^13,14^

Approaches that partly overcome the limited identification numbers nowadays typically employ either depletion of the most abundant proteins by selective binding to antibodies^15,16^ or the use of nanoparticles that form protein coronas, thereby enriching certain proteins by adsorption of high affinity binders.^17^ These nanoparticles are provided by numerous vendors and are available as mixtures of different entities that enable binding of various protein structures with different affinities.^18–22^ Other bead-based technologies are available that allow enrichment of extracellular vesicles.^23^ This class of nanovesicles is in common focus for biomarker discovery due to their origin from potentially any cell type or body fluid.^24^ As described just recently,^2,19^ the improvements in protein identifications with nanoparticle-based enrichment still cannot overcome initial shortcomings in blood sample generation. Contaminations arising from blood cellular material such as platelets, erythrocytes, or peripheral blood mononuclear cells (PBMCs) often lead to elevated protein ID numbers, compromising the identification of novel biomarkers. This can only be resolved by careful sample handling, appropriate centrifugation, and the prior selection of suitable blood collection tubes.

To date, sample preparation techniques have been primarily compared in HeLa cells^25^ or between a limited number of sample matrices and sample preparation techniques.^6,7^ In this study, we systematically compared proteomic profiles of five different blood matrices commonly used in clinical diagnostic settings. Serum, serum generated with a separation gel, and plasma anticoagulated with EDTA, citrate, or Li-heparin from three healthy voluntary donors were generated with standardized preanalytical measures, ensuring appropriate coagulation, centrifugation, and serum/plasma separation. Blood samples were pooled individually for each matrix type and processed by applying five commonly used sample preparation techniques. Trypsin digestion was performed exactly as described in the respective protocols and publications and varied between 2 h and overnight incubation at 37 to 47 °C. We measured all samples on two different LC–MS setups, namely, Q Exactive HF-X and timsTOF HT, thus covering a variety of instrumentation from rather low cost to the latest generation. Results indicate matrix- and sample preparation method-specific differences in the recovery of individual proteins, providing a valuable resource for future biomarker research.

## Experimental Procedure

### Experiment Design

Blood samples in the form of serum, serum prepared with a separation gel, EDTA plasma, citrate plasma, and Li-heparin plasma were consecutively obtained from the same three healthy individuals on the same day at the Institute of Laboratory Medicine, LMU University Hospital, LMU Munich, and were pooled after coagulation and centrifugation steps (2315*g*, 10 min, 20 °C) in equal ratios. Afterward, samples were aliquoted into 250 μL samples and stored at −80 °C. Provision and utilization of the human material were approved by the Ethics Committee of the Medical Faculty of LMU Munich (Reg. No. 17-012). The participants gave their written informed consent.

For sample preparation, varying amounts of serum or plasma were used, depending on the applied protocol. Protein concentrations of the utilized samples were determined by a colorimetric assay (Pierce BCA Protein Assay Kit, Thermo Scientific, Rockford, USA) and are specified in Supporting Information Table S1. For the commercial kits iST and ENRICH-iST, 1.25 and 20 μL, respectively, were used. For the perchloric acid workflow, 40 μL was used, and for the SPEED protocol, 2.5 μL of sample was used. The protein enrichment by SAX beads was performed with 40 μL of sample. Each sample preparation method was performed in technical replicates (*n* = 5) with all five blood matrix types on 1 day with a freshly thawed aliquot to avoid variation from freeze–thaw cycles.

### Sample Preparation

Samples were prepared manually according to the manufacturer’s protocol or the available preparation procedure.

#### iST

According to the manufacturer’s protocol, 50 μL of LYSE buffer was added to 1.25 μL of sample and then heated to 95 °C for 10 min at constant shaking with 1000 rpm on a thermal shaker. The samples were transferred to the iST cartridge, and 50 μL of digestion buffer was added. After incubation for 2 h at 37 °C with constant shaking at 500 rpm, the reaction was quenched by addition of 100 μL of STOP buffer. Samples were shaken for another 1 min at room temperature and 500 rpm to complete digestion termination. Peptide purification was performed on cartridges by washing the samples consecutively with 200 μL of two wash buffers (WASH1 and WASH2) with centrifugation steps between (2250*g* for 2 min). Peptide elution was conducted by addition of 100 μL of elute buffer following centrifugation at 2250*g* for 2 min. This step was repeated once, and the combined flow-through was aliquoted afterward into three parts (2 × 60 μL, 1 × 80 μL). Samples were dried by vacuum centrifugation and stored at −80 °C for further use.

#### ENRICH-iST

Sample preparation was conducted according to the manufacturer’s protocol. For this, magnetic beads were mixed thoroughly, aliquoted in portions of 25 μL, and washed individually three times by addition of 200 μL of EN-WASH buffer following incubation for 1 min at room temperature and shaking at 1200 rpm and subsequent magnetic separation. For the enrichment step, 80 μL of EN-BIND buffer and 20 μL of plasma or serum sample were added to the bead suspension in a reaction tube. After incubation for 30 min at 30 °C and constant shaking at 1200 rpm, beads were separated magnetically, the supernatant was discarded, and beads were washed three times with 100 μL of EN-BIND buffer. Lysis, digestion, and peptide purification were conducted as described in the iST protocol. Samples were dried by vacuum centrifugation and stored at −80 °C until further use.

#### perCA

Sample preparation with precipitation of large proteins by perchloric acid was conducted as described earlier.^26^ In short, 360 μL of water was added to 40 μL of plasma or serum, followed by addition of 20 μL of perchloric acid (70–72% p.a.). The suspension was vigorously agitated for 1 min and afterward incubated for 15 min at −20 °C. Precipitated proteins were pelleted by centrifugation for 60 min at 3200*g* at 4 °C. The pellet was discarded, and the supernatant was diluted with 1% (v/v) trifluoroacetic acid (TFA) in H_2_O. This solution was loaded onto a preequilibrated Oasis PRiME HLB μElution plate (Waters, no. 186008052) and washed with 200 μL 0.1% (v/v) TFA in H_2_O. Proteins were released by addition of 100 μL of elution buffer (90% (v/v) acetonitrile (MeCN), 9.9% (v/v) H_2_O, 0.1% TFA) and dried by vacuum centrifugation. After resuspension in 25 μL of 50 mM ammonium bicarbonate (ABC) buffer, 0.5 μg of trypsin was added, and samples were incubated overnight at 37 °C under constant mixing at 1000 rpm. Digestion was stopped by addition of 5 μL of 10% (v/v) formic acid (FA), and peptides were purified with stage tips and polystyrene–divinylbenzene reversed-phase sulfonate (SDBRPS) material. For this, stage tips were equilibrated in the following manner. A 100 μL portion of MeCN was added, and stage tips were centrifuged for 1 min at 800*g*. A 100 μL portion of 30% (v/v) MeOH, 1% (v/v) TFA, and 69% H_2_O was added, and stage tips were centrifuged for 3 min at 1000*g*. A 150 μL portion of wash buffer 3 (0.2% (v/v) TFA in H_2_O) was added, and stage tips were centrifuged to dryness for 1 min at 800*g*. Samples were diluted with 200 μL of loading buffer (1% (v/v) TFA in H_2_O) and were loaded onto equilibrated stage tips. Peptides were washed with 100 μL of wash buffer 1 (1% (v/v) TFA in EtOAc), 100 μL of wash buffer 2 (1% (v/v) TFA in *i*PrOH), and 150 μL of wash buffer 3. In between, samples were centrifuged for 3 min at 1000*g*. Peptides were eluted by addition of 60 μL of elution buffer (5% (v/v) NH_3_ in H_2_O, 15% (v/v) H_2_O, 80% (v/v) MeCN) and centrifugation for 2 min at 800*g*. Samples were dried by vacuum centrifugation and stored at −80 °C until further use.

#### SPEED

Sample preparation using the SPEED protocol was performed as described by Doellinger et al.^27^ with minor modifications. In brief, 2.5 μL of plasma or serum was incubated with 10 μL of TFA (100%) for 10 min at room temperature. Samples were neutralized by addition of 100 μL of 2 M Tris Base buffer before cysteine residues were reduced using 11.25 μL of 100 mM dithiothreitol (DTT) in H_2_O. Samples were alkylated with 12.4 μL of 400 mM iodoacetamide (IAA) in H_2_O and incubated at 95 °C for 5 min. Samples were diluted with 83.3 μL of a 10:1 mix of 2 M Tris Base and TFA and further diluted with 1 mL of H_2_O. Digestion was performed by addition of 3 μg of trypsin and incubation for 20 h at 37 °C with constant shaking at 600 rpm. Afterward, the reaction was quenched with 24 μL of TFA (100%), and half of the peptide solution was used for purification with SDBRPS stage tips as described above. Samples were stored at −80 °C until further use.

#### Mag-Net

Plasma protein enrichment using strong anion exchange (SAX) beads (MagReSyn SAX, ReSyn Biosciences) was performed using the Mag-Net protocol (version 5) as described earlier by Wu et al.^23^ A 12.5 μL portion of SAX beads per replicate was equilibrated by washing twice with 200 μL of wash buffer (50 mM bis-Tris propane, 150 mM NaCl, pH 6.3), gentle mixing for 30 s, and magnetic separation. A 40 μL portion of plasma or serum per replicate was diluted with an equal volume of bind buffer (100 mM bis-Tris propane, 150 mM NaCl, pH 6.3), gently mixed, and added to pre-equilibrated SAX beads. Samples were gently mixed for 30 min, and afterward, beads were separated magnetically. The supernatant was discarded, and high-abundance plasma proteins were depleted by three gentle washings with 500 μL of wash buffer. Vesicle lysis and cysteine residue reduction were performed by addition of 100 μL of buffer containing 50 mM Tris, 1% SDS, and 10 mM TCEP at pH 8.5. Samples were incubated for 60 min at 37 °C with gentle mixing before 3 μL of alkylating solution containing 15 mM IAA was added. After additional incubation for 30 min at 37 °C, protein capture was performed by addition of 240 μL of MeCN. Samples were mixed and incubated for 10 min at room temperature. After washing three times with 95% MeCN in H_2_O, on-bead digestion was performed with addition of 190 μL of ABC buffer (50 mM ammonium bicarbonate) and 10 μL of trypsin (50 ng/μL). Samples were incubated for 2 h at 47 °C with gentle mixing. Digestion was stopped by adding 10 μL of 10% TFA in H_2_O, and beads were magnetically removed. The supernatant was further used for cleanup by SDBRPS stage tips as described above. Afterward, samples were stored at −80 °C until further use.

#### MS Measurement

Each sample was measured on both LC–MS setups in the DIA mode. For measurement on Q Exactive HF-X and timsTOF HT, samples were dissolved in varying amounts of loading buffer (0.5% TFA, 2% MeCN, H_2_O; for further information, see Supporting Information Table 2).

#### Q Exactive HF-X

Peptides were injected on an UltiMate 3000 RSLC nano-HPLC (Dionex, Germering, Germany) coupled to a Q Exactive HF-X mass spectrometer (Thermo Fisher Scientific, Bremen, Germany) equipped with a nanoEase M/Z HSS T3 column (25 cm × 75 μm, C18 1.8 μm, 100 Å, Waters, Eschborn). Elution of peptides was performed in a 130 min run time with an 80 min nonlinear gradient and a column oven temperature of 40 °C. The flow rate was set to 250 nL/min with mobile phases A (0.1% FA, 2% MeCN, 97,9% H_2_O) and B (0.1% FA, MeCN). In detail, the gradient started at 3% B for 5 min and increased linearly in 80 min to 25% B, in 15 min to 40%, and in 5 min to 85% B. Composition with 85% B was held for 5 min, and then the fraction of B was reduced to 3% over 2 min and held at 3% B for a further 18 min. Mass spectrometric data were acquired with 1 MS scan followed by 37 fragmentation windows with varying isolation widths. Resolution of MS1 scans was set to 120,000 with a maximum ion-injection time of 120 ms and an AGC target of 3 × 10^6^. The scan range was set to 300–1650 *m*/*z*. Resolution on the MS2 level was set to 30,000 with a MS2 isolation width of 1350 *m*/*z*. Spray voltage was set to 1.5 kV with a capillary temperature of 250 °C.

#### timsTOF HT

Peptides were injected on an UltiMate 3000 RSLC nano-HPLC (Dionex, Germering, Germany) coupled to a timsTOF HT (Bruker, Bremen, Germany) equipped with a captive spray source with an Aurora Ultimate column (25 cm × 75 μm, C18 1.7 μm (AUR3-25075C18-CSI, IonOpticks, Australia)). Elution of peptides was performed in a 90 min run time with a 63 min gradient at a flow rate of 250 nL/min and a column oven temperature of 40 °C. Mobile phases A and B were composed in the same way as described for use with Q Exactive HF-X. The gradient started at a composition of 3% B for 5 min and increased linearly to 50% B in 63 min and afterward in 5 min to 98% B. 98% B was held for 4 min before the fraction of B was decreased to 3% in 1 min and held for an additional 12 min. Data were acquired in diaPASEF mode with a scan range of 100–1700 *m*/*z*, 16 PASEF windows, and a cycle time of 1.80 s. 1/*k*_0_ was set to 0.60–1.60 V·s/cm^2^ with a ramp time of 100 ms and a rolling average set to “on” (10×). Ion polarity was set to “positive”, the TIMS mode was enabled, and the collision energy was set to 10 eV. Glass capillary voltage was set to 4.5 kV with a 500 V end plate offset.

#### Data Analysis

Raw files were analyzed batchwise using DIA-NN (version 1.8.1) with an in silico generated spectral library predicted by DIA-NN (version 1.8.1) from a canonical human SwissProt fasta (20,440 entries, accession date: 28/09/2022). Trypsin/P was set as a protease with one allowed missed cleavage. Search parameters contained N-terminal methionine excision and cysteine carbamidomethylation as fixed modifications. The peptide length range was set to 7–30 amino acids, with a precursor charge allowed to be in the range of 1–4. The precursor *m*/*z* range was set to 300–1800 with a fragment ion *m*/*z* range of 200–1800. Mass accuracy was optimized data-dependently. Protein inference was performed on the gene level, the neural network classifier was set to “Single-pass mode”, and the quantification strategy was set to “Robust LC (high precision)”. Cross-run normalization was set to “RT-dependent”, and library generation was set to “Smart profiling”. Within each batch, MBR was enabled. For timsTOF HT data, the same parameters were set as for analysis of Q Exactive HF-X data except the mass accuracy settings were fixed at 10.0 ppm for MS1 and 20.0 ppm for MS2. DIA-NN output was automatically filtered for 0.01% FDR on the precursor and protein group level.

Downstream processing of data was performed using R (version 4.3) with the R package mpwR^28^ and Perseus^29^ (version 2.0.11.0). Protein groups were filtered based on at least 5 valid values in one group. For PCA analysis, missing values were imputed by a constant value equal to the lowest log2-transformed LFQ intensity observed.

## Results

In this study, we combined five different commonly used blood sample matrices with five commonly applied sample preparation methods in quintuplicate, measured on two individual LC–MS/MS setups, resulting in a total of 250 data files. Blood samples from three healthy individuals were pooled after appropriate coagulation and centrifugation steps (Figure 1). The commercially available iST-kit (Preomics) was considered as a neat plasma protein preparation and was compared to the SPEED protocol,^27^ which utilize concentrated trifluoroacetic acid for protein solubilization. Lately, bead-based approaches have gained substantial influence in plasma proteomics due to improved protein identifications; therefore, the Mag-Net protocol described by Wu et al.^23^ was applied, as well as another commercially available solution, ENRICH-iST. As a fifth approach, the perchloric acid workflow was utilized, which promises improved identification of low-abundance proteins by depletion through precipitation of high-abundance proteins.^26^ All samples were measured on two different LC–MS setups, a Q Exactive HF-X and a timsTOF HT, both coupled to Thermo Fisher UltiMate 3000 LC instruments.

**Figure 1:**
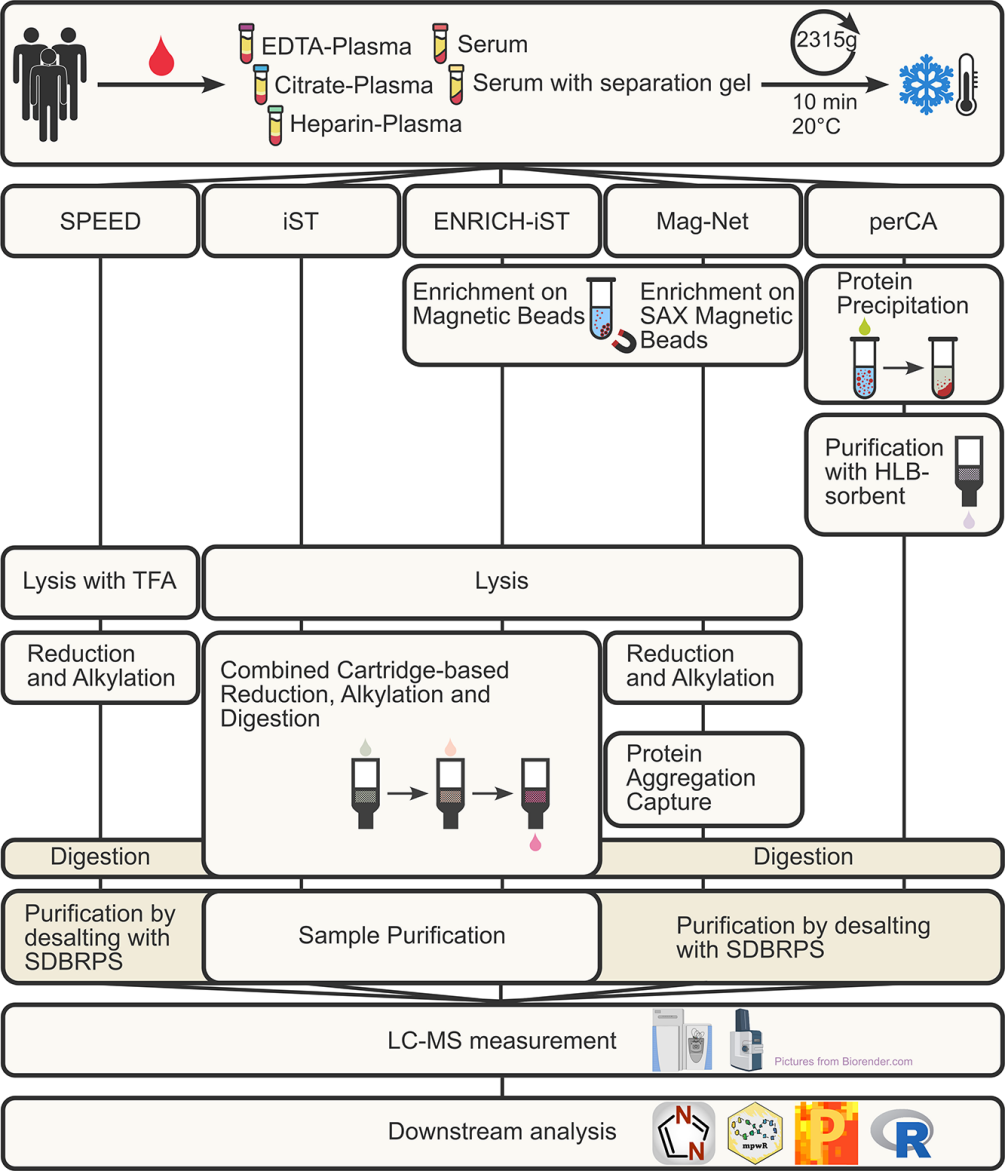
Experimental workflow for the sample preparation of five different blood matrices with methods SPEED, iST, ENRICH-iST, Mag-Net, and perchloric acid precipitation (perCA). Individual key steps of the different methods are depicted. Purified samples were measured on two instrument setups, and raw data were processed using DIA-NN 1.8.1 with further downstream processing using R, Perseus, and the software package mpwR. The figure was created with the help of Biorender.com.

### Protein Group Identification, Data Completeness, and Reproducibility

Protein group identification numbers on the timsTOF HT were higher compared to the data from the Q Exactive HF-X. Identification numbers range between 250 and 600 protein group IDs for Q Exactive HF-X and between 275 and 1100 for timsTOF HT (Figure 2A and Supporting Information Figure S1A). Among the five processed blood sample types measured on timsTOF HT, the sample preparation method Mag-Net returned the highest median protein group identification numbers between 747 and 1077. Perchloric acid (perCA) workflow generated the second highest numbers between 724 and 846, and preparation with ENRICH-iST resulted in IDs between 628 and 818. iST and SPEED as methods for neat plasma preparation gave the lowest ID numbers, ranging between 359 and 546 protein group IDs. While the identification rates among the blood sample types within the sample preparation method SPEED are relatively equal, differences are observable with all other preparation methods. In general, citrate anticoagulated plasma resulted in the lowest protein group identifications for all sample preparation techniques. EDTA plasma and heparinized plasma, as well as serum without gel separation, resulted in higher ID rates. The highest ID numbers could be achieved by using EDTA plasma with the Mag-Net workflow.

**Figure 2:**
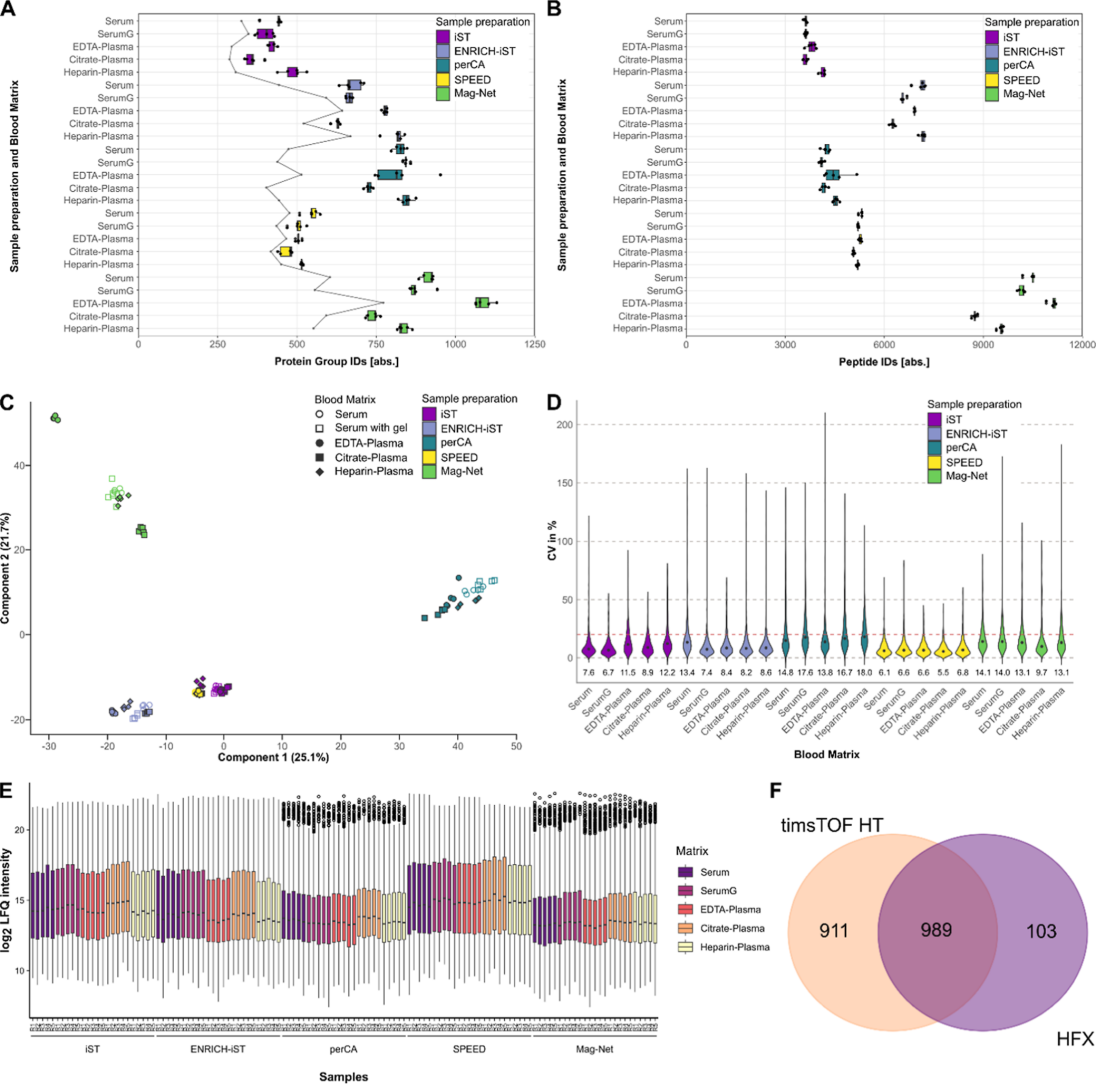
(A) Boxplot representation of protein group identifications from combinations of five sample preparations and five different blood matrices (5 technical replicates). The number of protein groups with CV values <20% on the protein group LFQ level is indicated by a gray line. (B) Boxplot representation of peptide identifications from combinations of five sample preparations and five different blood matrices. (C) Principal component analysis of filtered and log_2_-transformed LFQ intensities after imputation of missing values and normalization. (D) Violin plot representation of CV values based on protein group LFQ intensities. (E) Boxplot representation of log_2_-transformed LFQ intensities of individual sample sets for the timsTOF HT measurements. (F) Venn diagram depicting the overlap of protein group identifications of the same samples measured on timsTOF HT and Q Exactive HF-X.

Interestingly, the number of reproducibly quantifiable protein groups within a sample preparation method does not necessarily correlate with the overall number of identified protein groups. For example, between samples prepared with ENRICH-iST from serum or serum generated with a separation gel, there is a difference of almost 150 protein groups with a CV lower than 20%, while at the same time, almost the exact same median number of protein groups were identified (Figure 2A). A similar but less pronounced effect is also visible for both serum matrices prepared with the iST-kit. This could indicate that the separation gel has an ameliorating influence on sample quality. These trends are also recognizable in the data measured on the Q Exactive HF-X but are less pronounced due to the overall lower protein identifications.

On the peptide level, all sample preparation methods exceed 3000 peptides. With almost 9000 to more than 11,000 peptides, the Mag-Net workflow allowed the identification of by far the most peptides in our data set. The ENRICH-iST workflow led to the identification of 6200 to 7100 peptides, followed by the SPEED workflow with just over 5000 peptides. Interestingly, samples prepared with the perCA workflow yielded a similar number of peptides as samples prepared using the iST workflow while identifying more than twice as many protein groups. This effect can be explained by the substantially elevated number of protein groups with only one identified peptide in the perCA workflow compared to all other sample preparation methods, which might have a detrimental influence on the quantification of the proteins (Supporting Information Figure S2).

In a PCA analysis of normalized LFQ-intensity data, the samples clearly separate according to the sample preparation method (Figure 2C). Only the two neat sample preparation methods, iST and SPEED, do not separate considerably, which, interestingly, can be explained only to a limited extent by the proteins identified by applying these two methods, as only a small overlap of less than 40% can be observed in the pairwise comparison (Supporting Information Figure S3).

In general, median CVs for label-free quantification on the protein group level indicate good reproducibility for all sample measurements (Figure 2D). Values are well below 20% for most of the sample preparation types with SPEED exhibiting the lowest values between 5.6% and 6.8%. Both neat preparation protocols show good reproducibility, which could be partly explained by the lower number of protein group identifications. The perchloric acid workflow shows the highest distribution of CV values, which could be explained by the susceptibility to fluctuations in the precipitation step and the above-mentioned effect of the number of peptides per protein group. Using the modified version of the protocol as described in Albrecht et al.^31^ could potentially improve reproducibility. Non-normalized LFQ intensities of the protein groups show similar median values, indicating sufficient loading of the MS instrument. Only samples prepared by the Mag-Net workflow showed lower LFQ intensities, leaving room for optimization through higher injection volumes (Figure 2E).

### Pathway Enrichment by Specific Sample Preparation Techniques

After stringent filtering, the combination of all of the preparation methods identified by timsTOF HT resulted in a total of 1900 protein groups. Q Exactive HF-X identified 1092 protein groups in total, and the overlap between protein group identifications for both instruments is 989 protein groups (Figure 2F). Only 181 of the 1900 protein groups from the timsTOF HT data set are shared between all sample preparation techniques and blood matrices (Figure 3A). Except for protein C1QA, all proteins that are among the 28 most abundant proteins in human blood^32^ are included in the list of 181 protein groups.

**Figure 3:**
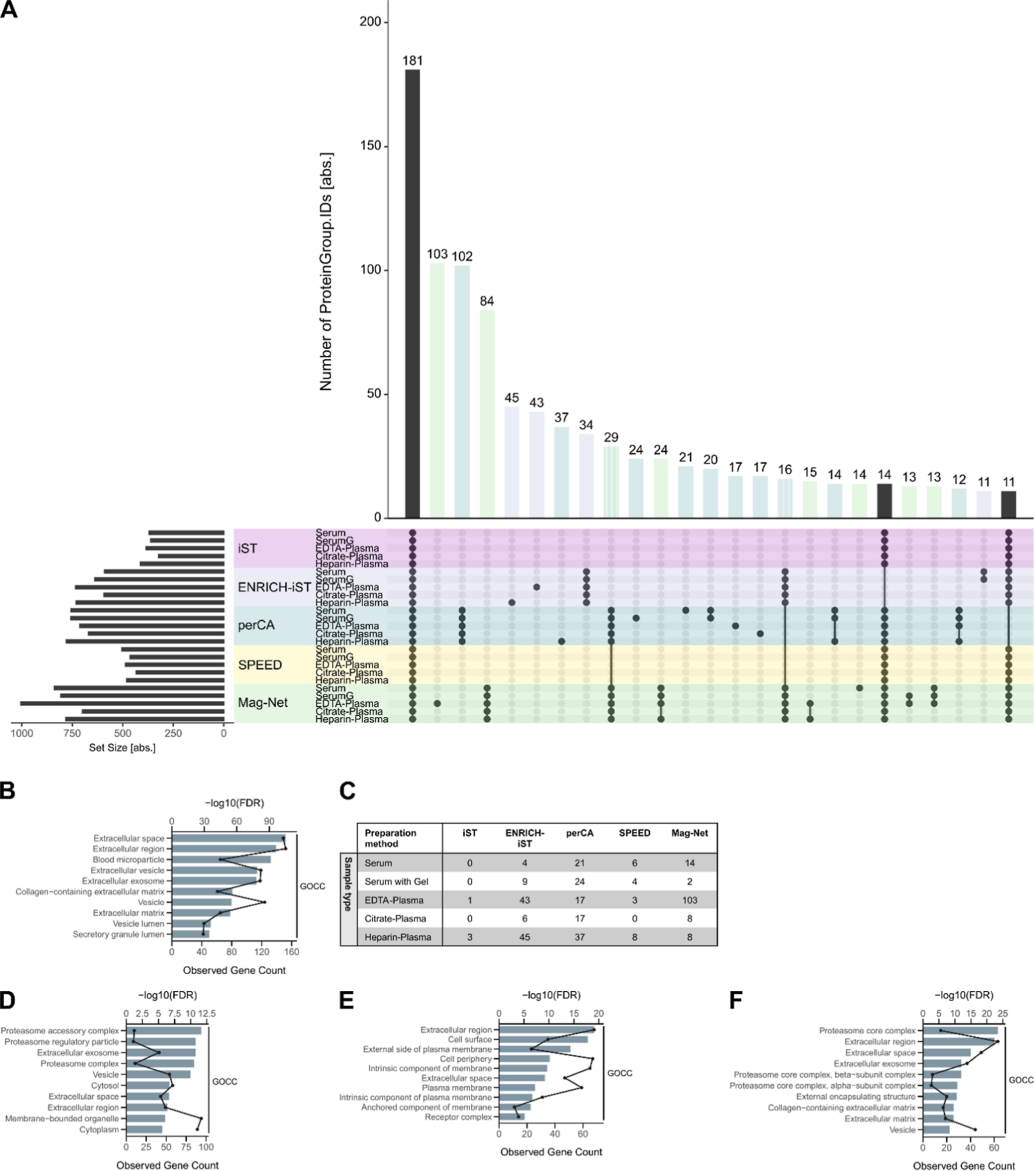
(A) Upset plot representation of overlapping protein group identifications from measurements on timsTOF HT. Visualization was set to only include clusters with more than 10 protein groups. (B) Tabular representation of individual clusters of protein groups that were identified by specific combinations of blood matrix and sample preparation method. (C–F) GOCC-term enrichment analysis of protein group clusters uniquely identified by (C) every sample preparation method in each blood matrix type and (D) Mag-Net workflow with EDTA plasma. (E) perCA-precipitation workflow with every blood matrix type and (F) Mag-Net workflow with every blood matrix type. Black lines indicate the observed gene count. A detailed list of enriched GO terms can be found in Supporting Information Table S5.

The highly abundant plasma proteins continue to be among the protein groups with the highest intensity, especially in the SPEED workflow. As expected, the LFQ intensities of the fibrinogens FBA, FBB, and FBG are lower in all serum samples compared to the plasma samples (Supporting Information Figures S5–S9). A prominent depletion of the highly abundant proteins is not observable in any of the sample preparations in our data set. This is illustrated by the log_2_-transformed LFQ intensities of the 28 high-abundance proteins indicated in the abundance rank plot (Supporting Information Figures S5–S9).

Moreover, 69 of the 71 protein groups that were found to be consistently measured throughout 8 different laboratories and LC–MS setups in a Germany-wide round robin study^13^ could be found in all blood matrices and sample preparation techniques (Supporting Information Table S3). The 181 proteins that were identified by timsTOF HT in every combination are involved in complement activation, stress response, and humoral immune response, as well as in the negative regulation of peptidase and hydrolase activity (Supporting Information Figure S10A). These proteins locate to a great extent to the extracellular region, blood microparticles, and vesicles (Figure 3B) and are characteristic of the functional constituents of the plasma proteome.

Obviously, subgroups were identified that are specific to certain sample preparation techniques or combinations with blood matrix types (Figure 3C), e.g., by the combinations EDTA plasma with Mag-Net (Figure 3D and Supporting Information Figure S10B), perCA with all blood matrices (Figure 3E and Supporting Information Figure S10C), and Mag-Net with all blood matrices (Figure 3F and Supporting Information Figure S10D). Interestingly, proteins that are involved in the cellular degradation processes, mainly proteasome subunits and ubiquitination-regulating proteins, were uniquely identified in EDTA plasma by the Mag-Net workflow (Supporting Information Figure S10B). By using the perCA workflow, proteins that are part of the immune system process and cell adhesion were uniquely identified (Supporting Information Figure S10C), which are mostly localized in the extracellular region, cell surface, or cell periphery (Figure 3E).

In all blood matrix types, the Mag-Net workflow not only enriched proteins of the proteasomal protein catabolic process and general proteolysis but also proteins that have molecular functions of glycosaminoglycan-, heparin-, sulfur compound-, integrin-, and calcium-binding proteins, indicating the unique enrichment of proteins that are involved in cell adhesion and signal transduction (Supporting Information Figure S10D).

Our data also show differences in the proteins that were exclusively enriched in heparin plasma or EDTA plasma by the ENRICH-iST protocol. However, in heparinized plasma, proteins involved in cadherin binding were enriched (Supporting Information Figure S11A); proteins of the glutathione metabolic process were exclusively enriched in EDTA plasma (Supporting Information Figure S11B). As expected, only a few proteins have been identified exclusively from neat plasma protocols. Similar results could be obtained from samples that were measured on Q Exactive HF-X (Supporting Information Figure S12).

In summary, we observed subgroups of uniquely identified protein groups for almost every combination of sample preparation technique and blood matrix type (Figure 3C). A detailed list of proteins that were uniquely identified with specific combinations of blood matrix type and sample preparation can be found in the Supporting Information (Table S4).

### Protein Abundance Is Dependent on the Sample Preparation Method and Blood Matrix

We next assessed how quantification of clinically relevant protein groups is influenced by different sample preparation methods and blood matrices. For this, we first evaluated the LFQ intensities of all identified protein groups and visualized these by hierarchical clustering (Figure 4). The abundance of protein groups can vary considerably within one sample preparation method, depending on the blood matrix type, as exemplified for selected protein sets in perCA, ENRICH-iST, and SPEED. We hypothesize that proteins may have different stabilities in blood samples depending on the applied anticoagulation reagent. Together with the utilized sample preparation protocol, this could result in varying detectability by LC–MS. If hierarchical clustering is restricted to the 181 overlapping proteins (Supporting Information Figure S13A), differences in LFQ intensity between blood sample types are still observable. These differences are prominent for the three fibrinogen proteins FGA, FGB, and FGG (Supporting Information Figure S13B). Due to the deliberate clotting process during sample preparation, serum samples naturally contain lower amounts of fibrinogens. Bead-based approaches like Mag-Net and ENRICH-iST can, however, enrich remaining FGG compared to neat workflows like iST, SPEED, and the perCA precipitation workflow. The influence of the utilized sample preparation method on identified protein abundance was substantial when examining the LFQ intensities of proteins FGA and FGB. The matrices serum and serum with a separating gel showed considerably reduced LFQ intensities for FGA and FGB in all sample preparation methods except in the perCA workflow (Supporting Information Figure S13B). A similar effect was visible when considering the acute-phase proteins haptoglobin (HP) and hemopexin (HPX). Especially HPX showed relatively similar LFQ-intensity profiles throughout all sample preparation techniques except for the Mag-Net workflow, illustrating the variable influence of sample preparation techniques (Supporting Information Figure S13C). Although variations arising from the manual sample preparation cannot be excluded, differences in LFQ intensity within the replicates of a sample preparation and blood matrix combination were substantial, especially for the highest abundant protein, albumin (Supporting Information Figure S13D). The SPEED workflow exhibited relatively homogeneous albumin levels over all blood matrix types, whereas the Mag-Net workflow again showed higher variations in a comparable pattern as with proteins HP and HPX. Although the blood samples were derived from healthy donors, the C-reactive protein (CRP) levels were considerably high (Supporting Information Figure S13E). This underpins the necessity for the use of device- and laboratory-specific standard values of this acute-phase protein and extends to sample preparation and blood matrix type.

**Figure 4:**
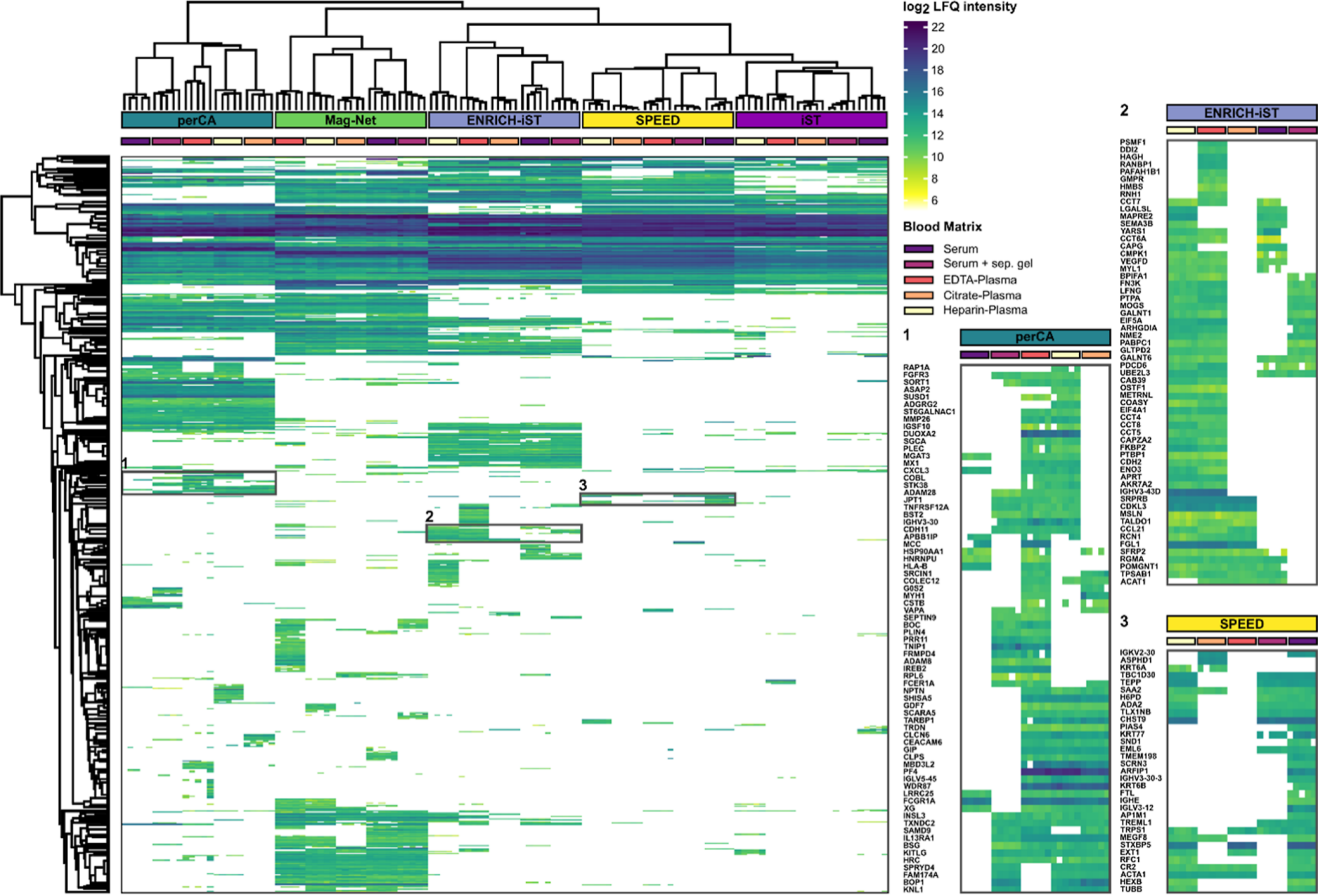
Hierarchical clustering of all identified protein groups. Differences in LFQ intensities of protein groups across the five utilized blood matrix types are exemplified for sample preparation methods perCA (1), ENRICH-iST (2), and SPEED (3).

Another preanalytical parameter that can have a substantial influence on the number of protein group identifications is the centrifugation speed at which serum or plasma was generated. Korff et al.^2^ could demonstrate a clear reciprocal correlation between centrifugation velocity and the number of platelet or erythrocyte markers contained. Accordingly, we also identified a panel of typical platelet markers (Supporting Information Figures S14 and S15), although we used a considerably high centrifugation speed of 2315*g*. This effect was most pronounced in bead-based enrichment methods such as ENRICH-iST and Mag-Net workflows, which, however, were also able to identify more protein groups overall. Platelet-specific marker PPBP, on the other side, was most intense in the perCA workflow, which is in accordance with previously reported data. Neat workflows reported overall lower numbers of platelet-specific markers, presumably due to an overall lower number of protein identifications.

### Mag-Net Workflow Uniquely Enriched Subunits of the Proteasome

GO-term enrichment analysis revealed a Mag-Net workflow-specific enrichment and identification of members of the proteasome core complex as well as subunits of the proteasome regulatory particle. We observed that almost all subunits of the human proteasome were identified by specific combinations of sample preparation techniques and the blood matrix. The 26S proteasome consists of the 20S protease core complex and two 19S regulatory subunits, which are responsible for recognition of poly ubiquitinated substrate proteins as well as for unfolding and translocation of proteins that are destined for proteasomal degradation.^33,34^ The proteolytic core unit, consisting of four stacked heptameric rings composed of two rings of α and β subunits, respectively, was identified by the Mag-Net workflow in all blood matrices (Figure 5). Only subunit PSMB7 was not detected in the citrate plasma. Interestingly, subunit PSMA2 was also detected in serum with the iST workflow, and PSMA3 was detected in all matrices except heparin plasma with the perCA precipitation workflow.

**Figure 5:**
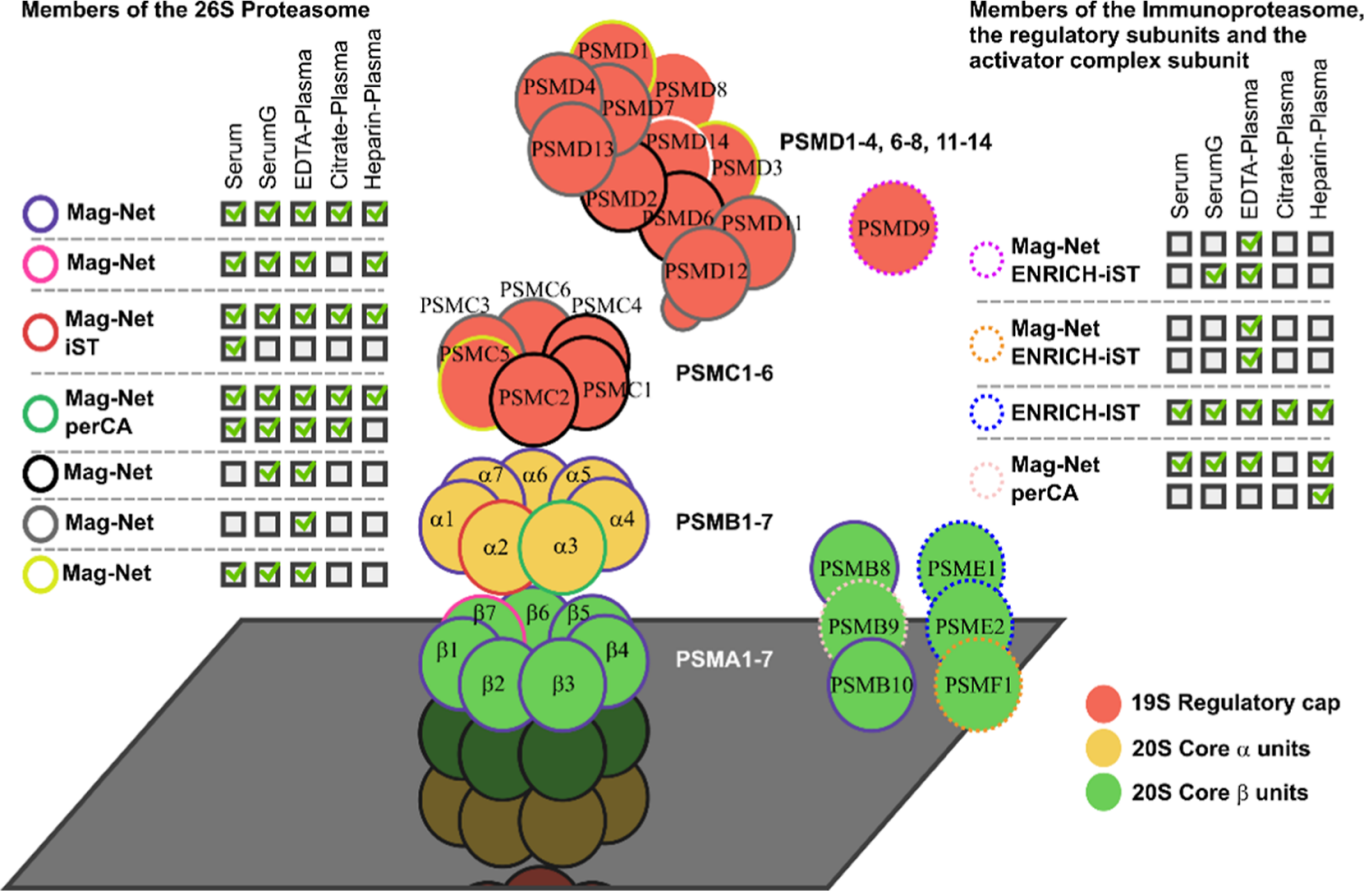
Graphical representation of proteasomal subunits and their identification by individual combinations of sample preparation procedures and blood matrices. Only one-half of the σ_h_-symmetric proteasome is visualized. Subunits that were identified are color-coded based on the applied sample preparation method and blood matrix.

We further observed that almost all members of the regulatory subunit were identified when samples are prepared from EDTA plasma with the Mag-Net workflow. While proteins PSMC3, PSMC6, PSMD4, PSMD7, PSMD11, PSMD12, and PSMD13 were exclusively identified in the samples from EDTA plasma with the Mag-Net workflow, subunits PSMC1, PSMC2, PSMC4, PSMD2, and PSMD6 were additionally found in serum with separation gel. Subunits PSMC5, PSMD1, and PSMD3 were found in serum with and without separation gel as well as in EDTA plasma. Subunits PSMD8 and PSMD14 were not observed in our data set.

In situations of oxidative stress, the transcription of additional proteasome subunits can be triggered by interferon-γ (IFN-γ). Replacement of subunits PSMB5, PSMB6, and PSMB7 leads to the formation of the immunoproteasome, which specifically produces smaller peptides for presentation on the cell surface by the MHC 1 complex.^35^ We observed that two of these three immunoproteasome subunits, PSMB8 and PSMB10, can be identified in all blood matrix types using the Mag-Net workflow. PSMB9, however, could not be observed in citrate plasma applying the Mag-Net workflow but could in heparin plasma with perCA precipitation. The 20S proteasome can also form complexes with other regulatory subunits. The best-known additional regulatory complexes are formed by the paralogues PSME1, PSME2, and PSME3, which constitute either heteromeric (PSME1/PSME2) or homomeric (PSME3) rings. These bind to the apical site of the proteasome core complex and fine-tune the protein degradation for MHC class I antigen presentation.^36^ Our experimental data again showed a specific representation of the PSME1/PSME2 complex, this time in all samples that were processed by ENRICH-iST. The proteasome inhibitor PSMF1 was found by either ENRICH-iST or the Mag-Net workflow solely in EDTA plasma. The subunits PSMD8 and PSMD14 of the regulatory particle are absent in our data set, as well as the sperm-specific proteasomal subunit PSMA8 and the thymoproteasome subunit PSMB11. The latter two subunits have so far not been described by the human protein atlas as being present in blood.

## Discussion

In this study, we systematically investigated the influence of clinically relevant blood sample types on the number and identity of detectable proteins from human blood. We further assessed the reproducibility and data completeness of different sample preparation methods. Since in laboratory medicine and biobanks patient blood is often available with various types of anticoagulants or as serum prepared with and without separation gel, we included five different clinically relevant blood matrices in our study. We found that citrate plasma yielded the least protein identifications of all five blood matrices in every sample preparation method on both applied mass spectrometry instruments. Heparin plasma performed best for commercially available kits from Preomics, while EDTA plasma gave the most protein group IDs for the Mag-Net workflow. This finding is to be expected as most commercial workflows recommend the use of plasma or are optimized on an EDTA plasma. The considerable difference of over 300 protein group identifications between EDTA plasma and citrate plasma emphasizes the necessity of using suitable blood matrices for mass spectrometric analysis. Since strong anion-exchange magnetic microparticles from ReSyn BioSciences are discussed to enrich extracellular vesicles, we speculate that these nanosized particles induce varying stability of EVs in different anticoagulated plasma types. Furthermore, we could confirm the results of an earlier study^2^ in which neat workflows and the perCA precipitation approach are less influenced by different blood matrices. Especially in the Mag-Net workflow, the same trend in the number of protein group identifications is observed, with EDTA plasma yielding the highest number, followed by serum (with and without separation gel) and heparinized plasma.

In our hands, the SPEED workflow exhibited the highest reproducibility among all blood matrix types but at the same time also returned the second lowest protein group identification numbers. In general, neat plasma preparation techniques revealed the lowest identification numbers of 359 to 546 protein groups, which is in accordance with previous experimental findings.^13^

181 protein groups were identified with every combination of the sample preparation method and blood matrix. In this subset, all but one of the 28 most abundant plasma proteins^32^ can be found. At the same time, most of the 181 protein groups greatly differ in their abundance, depending on the sample preparation techniques. We speculate that the major reason for this might be the solubilization or precipitation of the proteins and the varying affinity toward the nanoparticles in the bead-based workflows.

Comparison of the individually identified protein groups revealed that subsets of the plasma proteome could be made accessible by different sample preparation techniques. GO-term enrichment analysis revealed that the Mag-Net workflow selectively enriches components of the 26S proteasome. Whether these proteasomal subunits originate from platelets or are part of the extracellular circulating proteasomes remains arguable since the Mag-Net workflow enriches extracellular vesicles, and we observe some marker proteins for platelet contamination, even though the centrifugation velocity in the clinical SOP was comparatively high (2315*g*) (Supporting Information Figures S14 and S15).

Detection of proteasome subunits has not yet been discussed in the context of plasma proteomic protocols but could be helpful, as increased levels of extracellular circulating proteasomes have already been observed in patients with various cancers. c-Proteasomal presence in serum and plasma was previously investigated as a biomarker for cancerous malignancies such as adult T cell leukemia,^37^ hepatocellular carcinoma,^37,38^ metastatic melanoma,^39^ and a variety of systemic autoimmune diseases,^40^ to only name a few.

Our study faces several limitations. First, the overall identification rate of bead-based technologies falls short of expectations from previous studies. This could be related to the comparatively high centrifugation speed used in the clinical routine SOP protocol for matrix extraction. The applied centrifugation speed of 2315*g* is considerably higher than in the collection protocols often recommended for bead-based enrichment studies (1500*g*) and should deliver matrices with comparably lower platelet contaminations. Additionally, the sensitivity and performance of the MS instruments used may be a limitation, potentially inferior to those of the currently most powerful devices. Furthermore, the in silico generated library utilized can have a considerable influence on the number of protein groups identified. We decided to use a stringent approach based on a FASTA library with canonical protein sequences even if this means that fewer protein groups are identified. The use of automation could additionally increase the reproducibility.

Future experiments will include blood samples from individual donors to highlight further differences and to make protein-specific recommendations for large cohorts.

In conclusion, by comparing five different clinically relevant blood matrix types combined with five common sample preparation techniques, we identified clusters of protein groups that were unique to specific combinations of blood matrix and sample preparation methods. This study could serve as a useful resource for the investigation of future protein biomarkers by providing suggestions for the selection of appropriate combinations of sample preparation techniques and blood matrices.

This article contains supplemental information and Supporting Information Tables S1–S5.

## Supplementary Material









## Data Availability

Mass spectrometric data have been deposited to the ProteomeXchange Consortium via the PRIDE^30^ partner repository under the data set identifier PXD066304.

## Notes

Supplementary Table 1. Protein concentration of blood samples; Supplementary Table 2. Sample preparation parameters for LC–MS measurement; Supplementary Figure 1. Boxplot representing protein group identifications for Q Exactive HF-X, boxplot representation of peptide identifications, principal component analysis, violin plot representation of CV values, and boxplot representation of log2-transformed LFQ intensities of individual sample sets; normalized mean peptide count per protein group; Supplementary Figure 3. Pairwise overlap of identified protein groups for all 125 replicates from measurement on timsTOF HT; Supplementary Figure 4. Pairwise overlap of identified protein groups for all 125 replicates from measurement on Q Exactive HF-X; Supplementary Figure 5. Rank plots of sample preparation iST and all blood matrix types; Supplementary Figure 6. Rank plots of sample preparation ENRICH-iST and all blood matrix types; Supplementary Figure 7. Rank plots of sample preparation perCA and all blood matrix types; Supplementary Figure 8. Rank plots of sample preparation SPEED and all blood matrix types; Supplementary Figure 9. Rank plots of sample preparation Mag-Net and all blood matrix types; Supplementary Figure 10. GOBP- and GOMF-term enrichment analysis of protein group clusters in data sets from timsTOF HT; Supplementary Figure 11. Additional GO-term enrichment analysis of protein group clusters in data sets from timsTOF HT; Supplementary Figure 12. Upset plot representation of overlapping protein group identifications from measurement on Q Exactive HF-X and identification of individual clusters of protein groups by specific combinations of blood matrix and sample preparation method, with clusters depicted in tabular representation; Supplementary Figure 13. Heatmap representation of non-normalized, log2-transformed LFQ intensities and profile plots for selected protein groups; Supplementary Figure 14. Non-normalized LFQ intensities of known platelet markers from measurements on timsTOF HT in heatmap representation; Supplementary Figure 15. Non-normalized LFQ intensities of known platelet markers from measurements on Q Exactive HF-X in heatmap representation (PDF)
Log-transformed LFQ intensities of timsTOF HT data for protein groups identified in the longitudinal study (XLSX)
Individual protein groups for measurement on timsTOF HT (XLSX)
GO-term enrichment analyses for data generated on timsTOF HT (XLSX)

## Abbreviations

ABC: ammonium bicarbonate
AGC: automatic gain control
BCA: bicinchoninic acid
CV: coefficient of variation
DIA: data-independent acquisition
DTT: dithiothreitol
EtOAc: ethyl acetate
FA: formic acid
FDA: Food and Drug Administration
FDR: false discovery rate
GO: gene ontology
HPLC: high-performance liquid chromatography
HLB: hydrophilic–lipophilic-balance
IAA: iodoacetamide
ID: identification
iPrOH: iso-propanol
iST: in-stage tip
LC-MS/MS: liquid chromatography–mass spectrometry
LFQ: label-free quantification
LMU: Ludwig-Maximilians-Universität München
MeCN: acetonitrile
MeOH: methanol
NH3: ammonia
PASEF: parallel accumulation serial fragmentation
PCA: principal component analysis
PBMC: peripheral blood mononuclear cell
perCA: perchloric acid
SAX: strong-anion exchange
SDBRPS: polystyrene-divinylbenzene reversed-phase sulfonate
SDS: sodium dodecyl sulfate
SOP: standard operating procedure
SPEED: Sample Preparation by Easy Extraction and Digestion
TCEP: tris­(2-carboxyethyl)-phosphine-hydrochloride
TFA: trifluoroacetic acid
TRIS: *tris*(hydroxymethyl)-aminomethane
