# Supplementary material for: Blood Matrices and Sample Preparation Influence Blood Marker Discovery
Source: J Proteome Res. 2025 Dec 16;25(1):405–17. doi: 10.1021/acs.jproteome.5c00836 (PMC12772121; doi:10.1021/acs.jproteome.5c00836)
Supplement: Supplementary file 1 [file pr5c00836_si_001.pdf]

Supporting Information

Blood matrices and sample preparation influence blood marker discovery

Thomas F. Gronauer[1], Juliane Merl-Pham[1], Christine von Toerne[1], Katharina Habler[2], Daniel Teupser[2], Stefanie M. Hauck*[1]

[1]Metabolomics and Proteomics Core (MPC), Helmholtz Zentrum München, German Research Center for Environmental Health (GmbH), 80939, Munich, Germany

[2]Institute of Laboratory Medicine, LMU University Hospital, LMU Munich, 81377, Munich, Germany

* Corresponding Author: Stefanie M. Hauck - Metabolomics and Proteomics Core (MPC), Helmholtz Zentrum München, German Research Center for Environmental Health (GmbH), 80939, Munich, Germany; orcid.org/0000-0002-1630-6827; E-Mail: stefanie.hauck@helmholtz-munich.de

Contents

Supplementary Figures ... 3

Protein concentration of blood samples ... 3

Sample preparation parameters ... 3

Additional data from measurements on Q Exactive HF-X ... 4

Normalized mean peptide count per protein group ... 5

Pairwise overlap between datasets for timsTOF HT data ... 6

Pairwise overlap between datasets for Q Exactive HF-X data ... 7

Rank plots for timsTOF HT data prepared with iST kit ... 8

Rank plots for timsTOF HT data prepared with ENRICH-iST kit ... 9

Rank plots for timsTOF HT data prepared with perCA protocol ... 10

Rank plots for timsTOF HT data prepared with SPEED-protocol ... 11

Rank plots for timsTOF HT data prepared with MagNet protocol ... 12

GO-term enrichment analysis for timsTOF HT data ... 13

Additional GO-term enrichment analysis for timsTOF HT data ... 14

Upset plot and GO-term enrichment analysis of Q Exactive HF-X data ... 15

Comparison of log_2-transformed LFQ-intensities for timsTOF HT data ... 16

LFQ-intensities of known platelet markers for timsTOF HT data ... 17

LFQ-intensities of known platelet markers for Q Exactive HF-X data ... 18

Supplementary Table 3: log-transformed LFQ-intensities of timsTOF HT data for protein groups identified in the longitudinal study, Supplementary_Table_3_LT-comparison.xlsx

Supplementary Table 4: Individual protein groups for measurement on timsTOF HT, Supplementary_Table_4_Individual_protein_groups.xlsx

Supplementary Table 5: GO-term enrichment analyses for data generated on timsTOF HT, Supplementary_Table_5_GO-term_enrichment_analysis_timsTOFHT.xlsx

Supplementary Figures

Protein concentration of blood samples

Supplementary Table 1. Protein concentration of blood samples.

Sample type	Protein conc. [µg/µL]
Serum	71.3
Serum with separation gel	70.4
EDTA-Plasma	70.2
Citrate-Plasma	56.4
Heparin-Plasma	59.8

Sample preparation parameters

Supplementary Table 2. Sample preparation parameters for LC-MS measurement.

	Q Exactive HFX		timsTOF HT	
Sample prep type	Volume for dissolving peptides [µL]	Injection volume [µL]	Volume for dissolving peptides [µL]	Injection volume [µL]
iST	40	1.5	40	1
ENRICH-iST	15	1	15	1.5
perCA	40	8	20	5
SPEED	80	1	40	1
Mag-Net	15	1	15	1.5

Additional data from measurements on Q Exactive HF-X

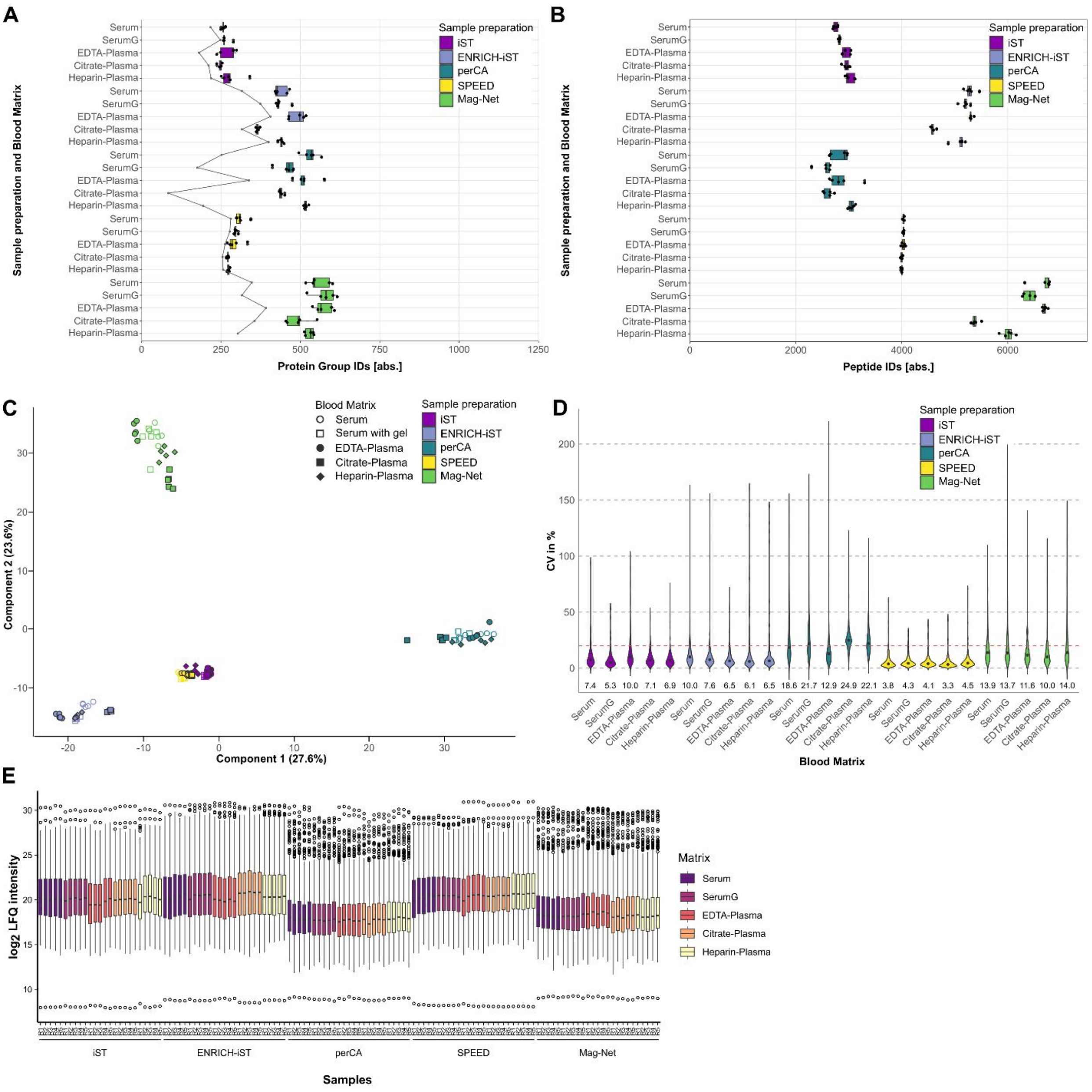

__Supplementary Figure 1__. Results from measurements on Q Exactive HF-X. (A) Boxplot representation of protein group identifications from combinations of five sample preparation methods and five different blood matrices (5 technical replicates). Number of protein groups with CV-values < 20% on protein group LFQ-level are indicated by a grey line. (B) Boxplot representation of peptide identifications from combinations of five sample preparations and five different blood matrices. (C) Principal component analysis of filtered and log$_2$-transformed LFQ-intensities after imputation of missing values and normalization. (D) Violin plot representation of CV-values based on protein group LFQ-intensities. (E) Boxplot representation of log$_2$-transformed LFQ-intensities of individual sample sets for the Q Exactive HF-X measurements.

Normalized mean peptide count per protein group

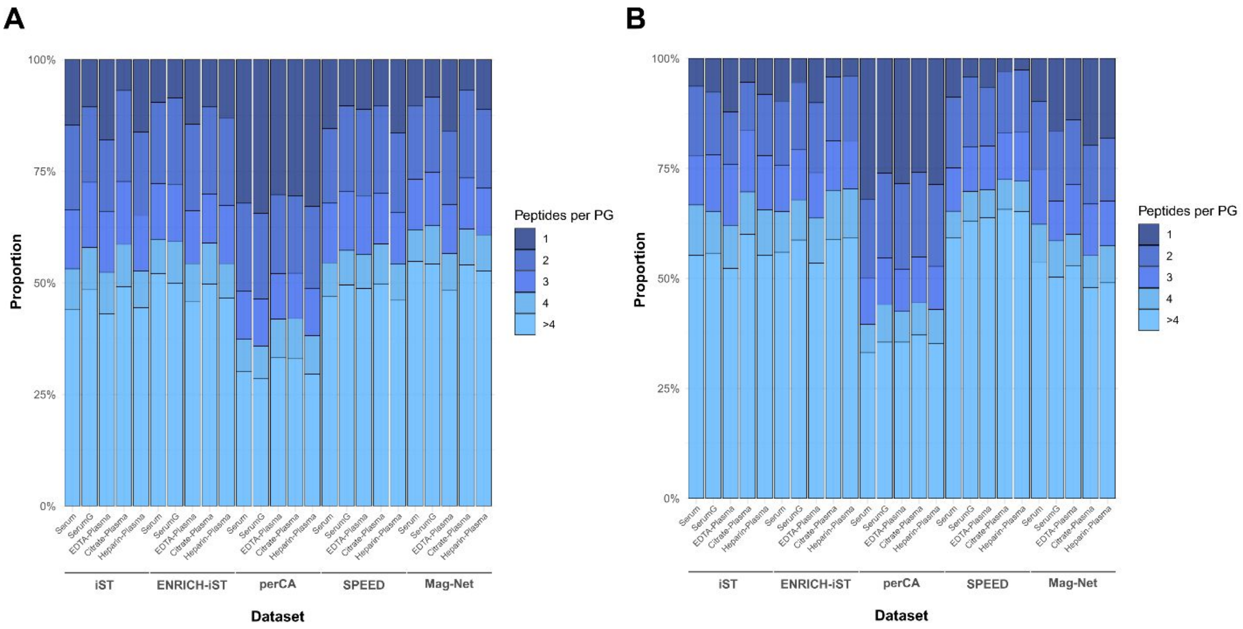

Supplementary Figure 2*. Normalized mean peptide count per protein group for all combinations of blood matrix and sample preparation technique for (A) timsTOF HT and (B) Q Exactive HF-X.*

Pairwise overlap between datasets for timsTOF HT data

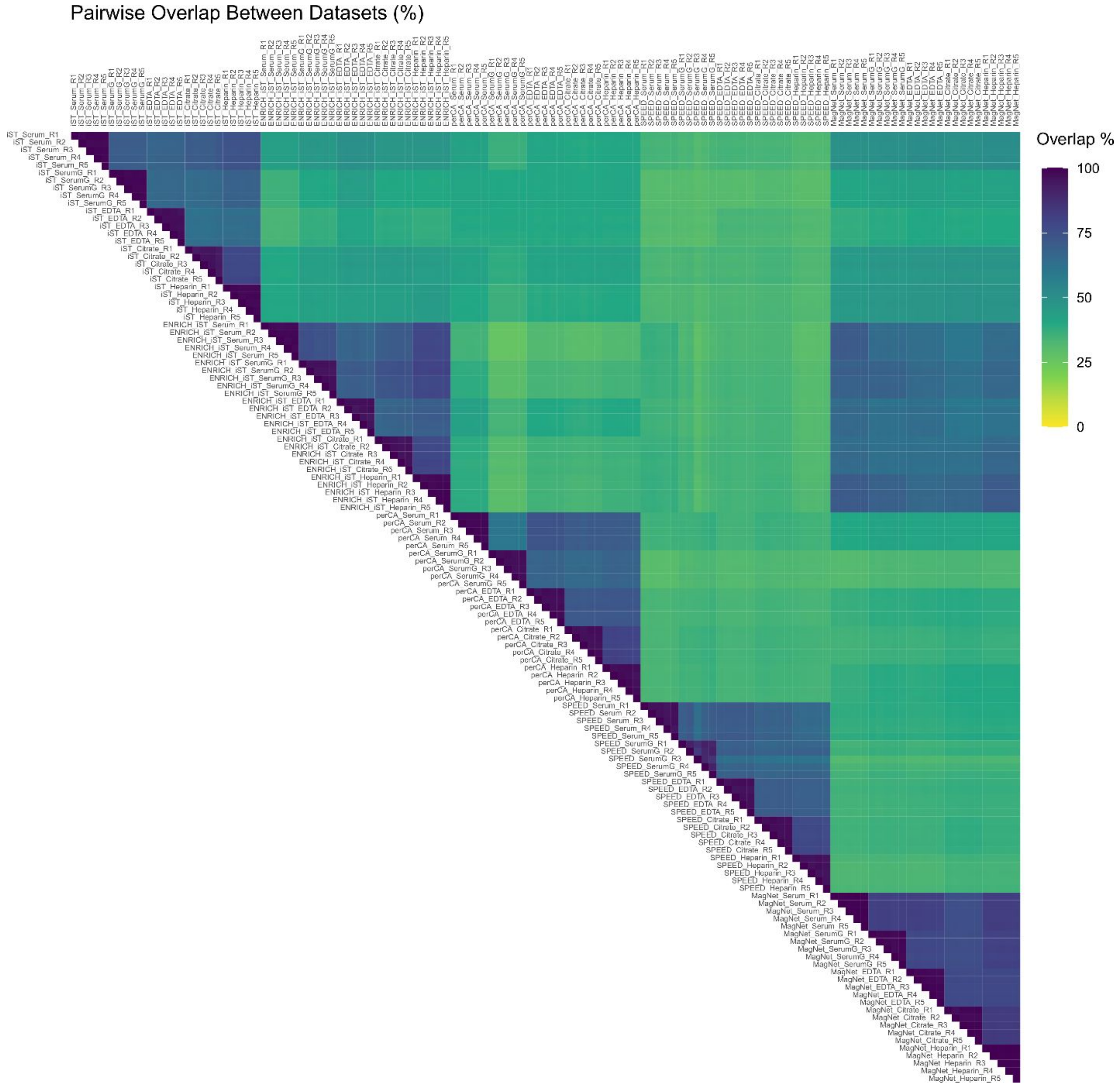

***Supplementary Figure 3**. Pairwise overlap of identified protein groups for all 125 replicates from measurements on timsTOF HT.*

Pairwise overlap between datasets for Q Exactive HF-X data

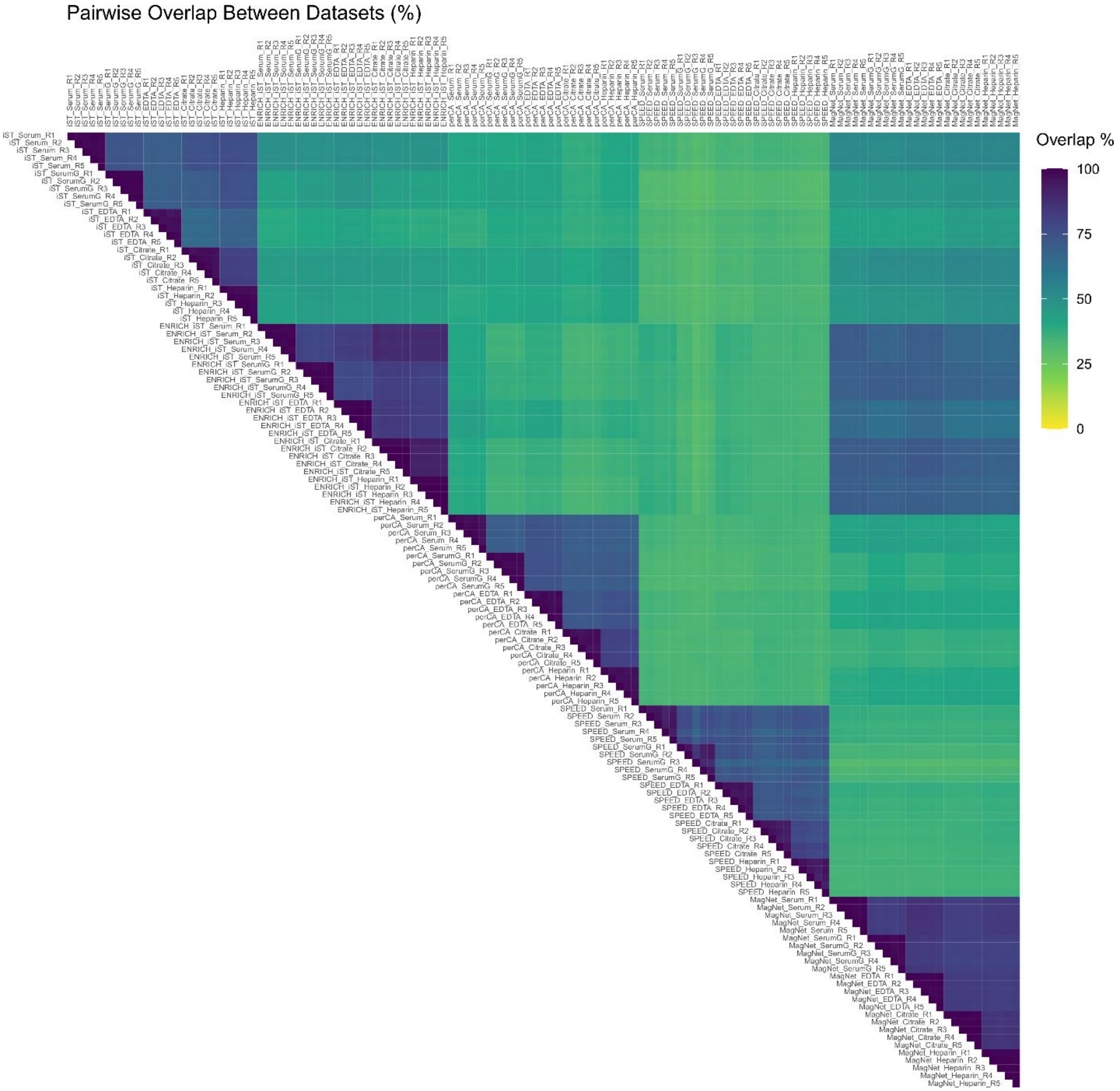

Supplementary Figure 4*. Pairwise overlap of identified protein groups for all 125 replicates from measurements on Q Exactive HF-X.*

Rank plots for timsTOF HT data prepared with iST kit

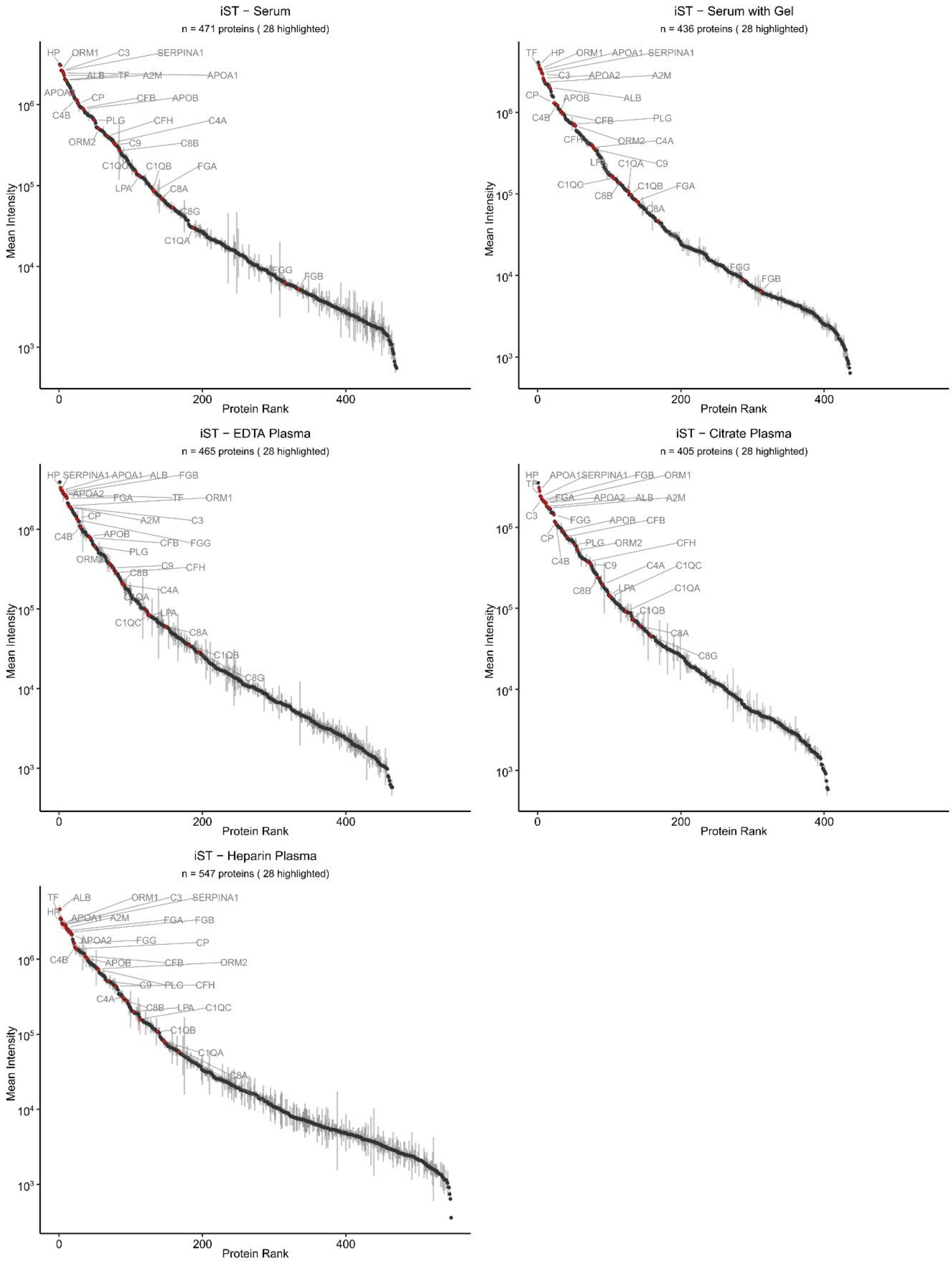

Supplementary Figure 5. *Rank plots of the combination of sample preparation method iST and all blood matrix types. Mean values from five replicates of protein group LFQ intensities are plotted in decreasing order. The 28 most abundant proteins are highlighted in red and labeled with their gene name.*

Rank plots for timsTOF HT data prepared with ENRICH-iST kit

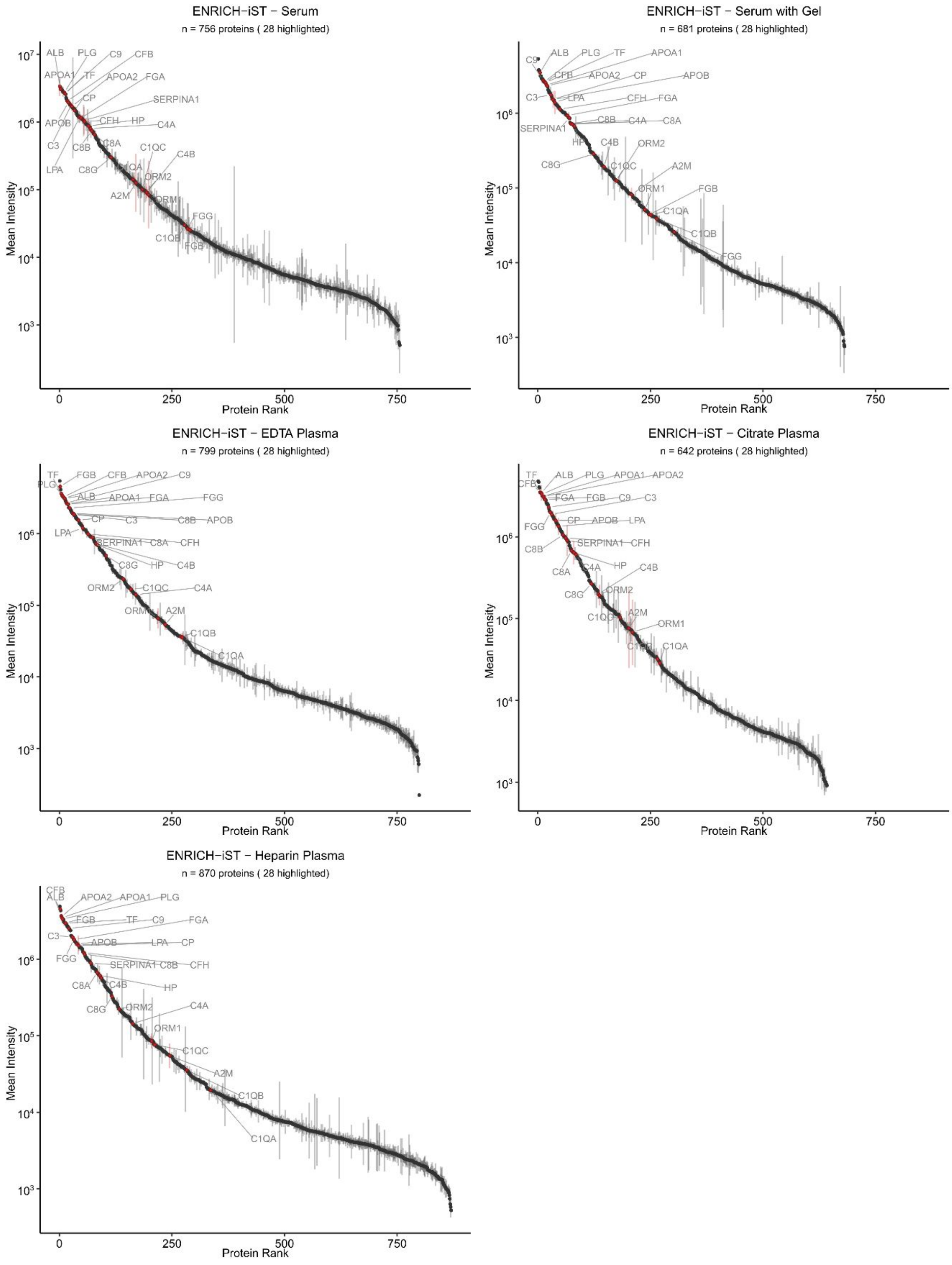

Supplementary Figure 6*. Rank plots of the combination of sample preparation method ENRICH-iST and all blood matrix types. Mean values from five replicates of protein group LFQ intensities are plotted in decreasing order. The 28 most abundant proteins are highlighted in red and labeled with their gene name.*

Rank plots for timsTOF HT data prepared with perCA protocol

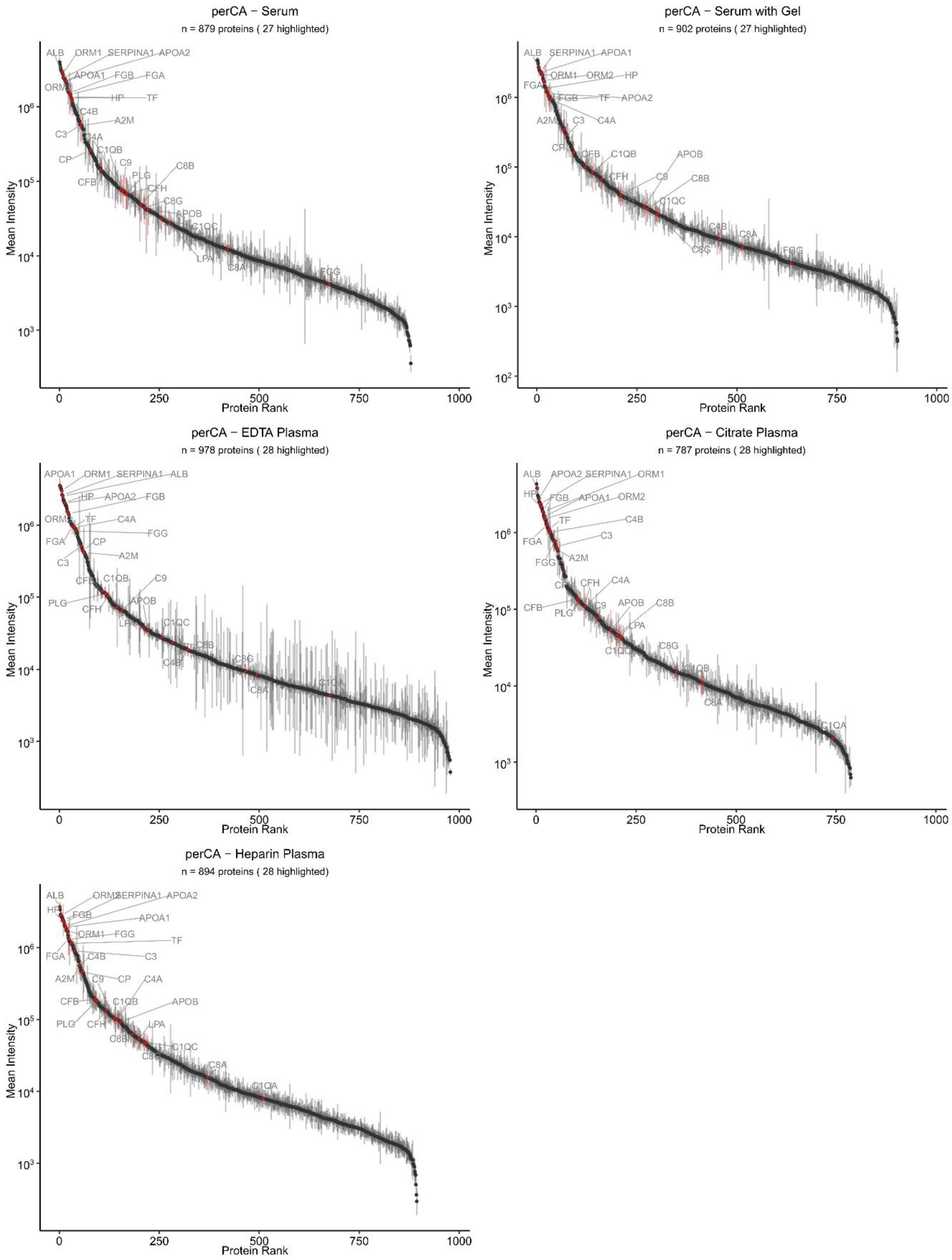

Supplementary Figure 7*. Rank plots of the combination of sample preparation method perCA and all blood matrix types. Mean values from five replicates of protein group LFQ intensities are plotted in decreasing order. The 28 most abundant proteins are highlighted in red and labeled with their gene name.*

Rank plots for timsTOF HT data prepared with SPEED-protocol

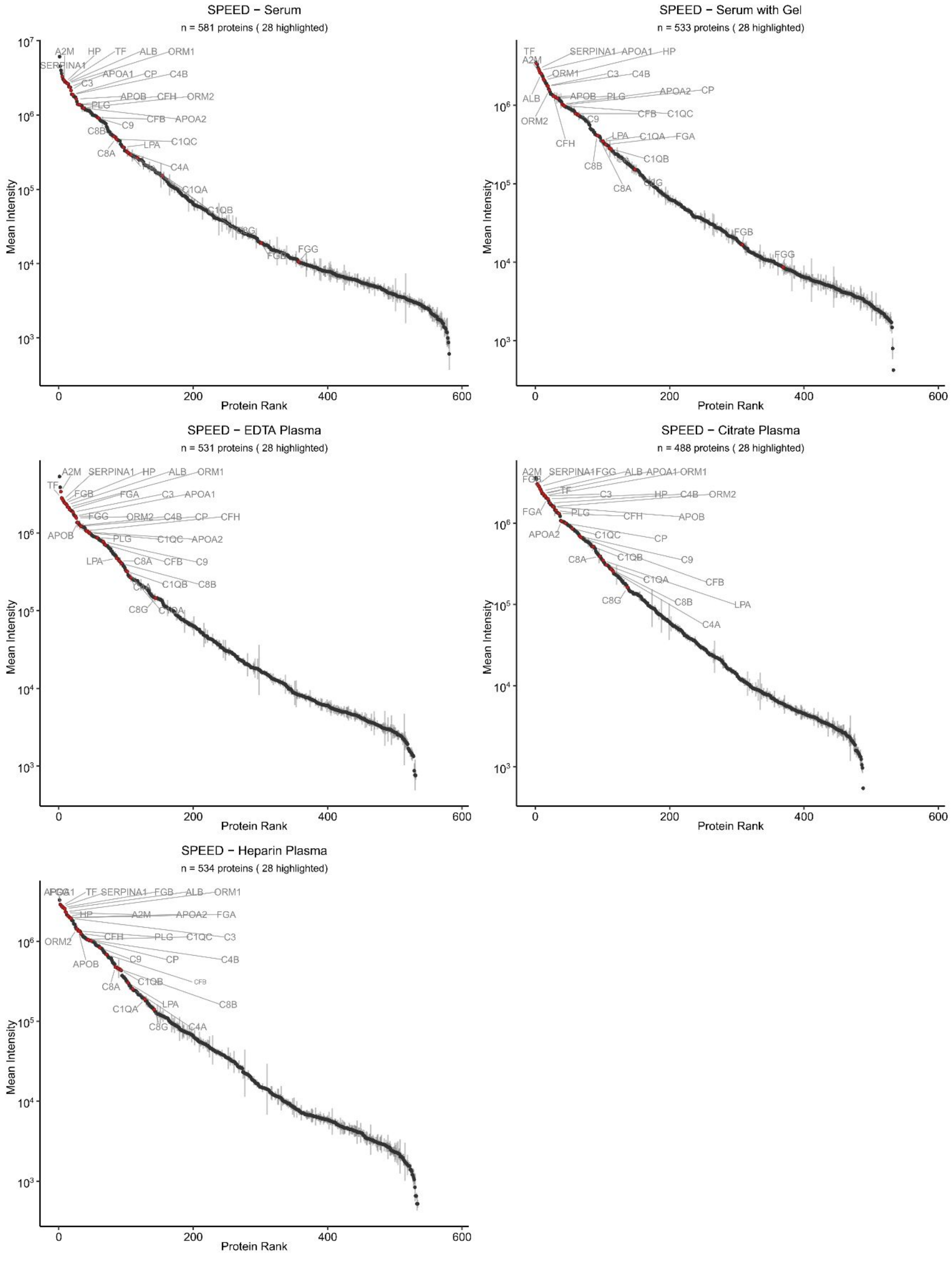

__Supplementary Figure 8__. Rank plots of the combination of sample preparation method SPEED and all blood matrix types. Mean values from five replicates of protein group LFQ intensities are plotted in decreasing order. The 28 most abundant proteins are highlighted in red and labeled with their gene name.

Rank plots for timsTOF HT data prepared with MagNet protocol

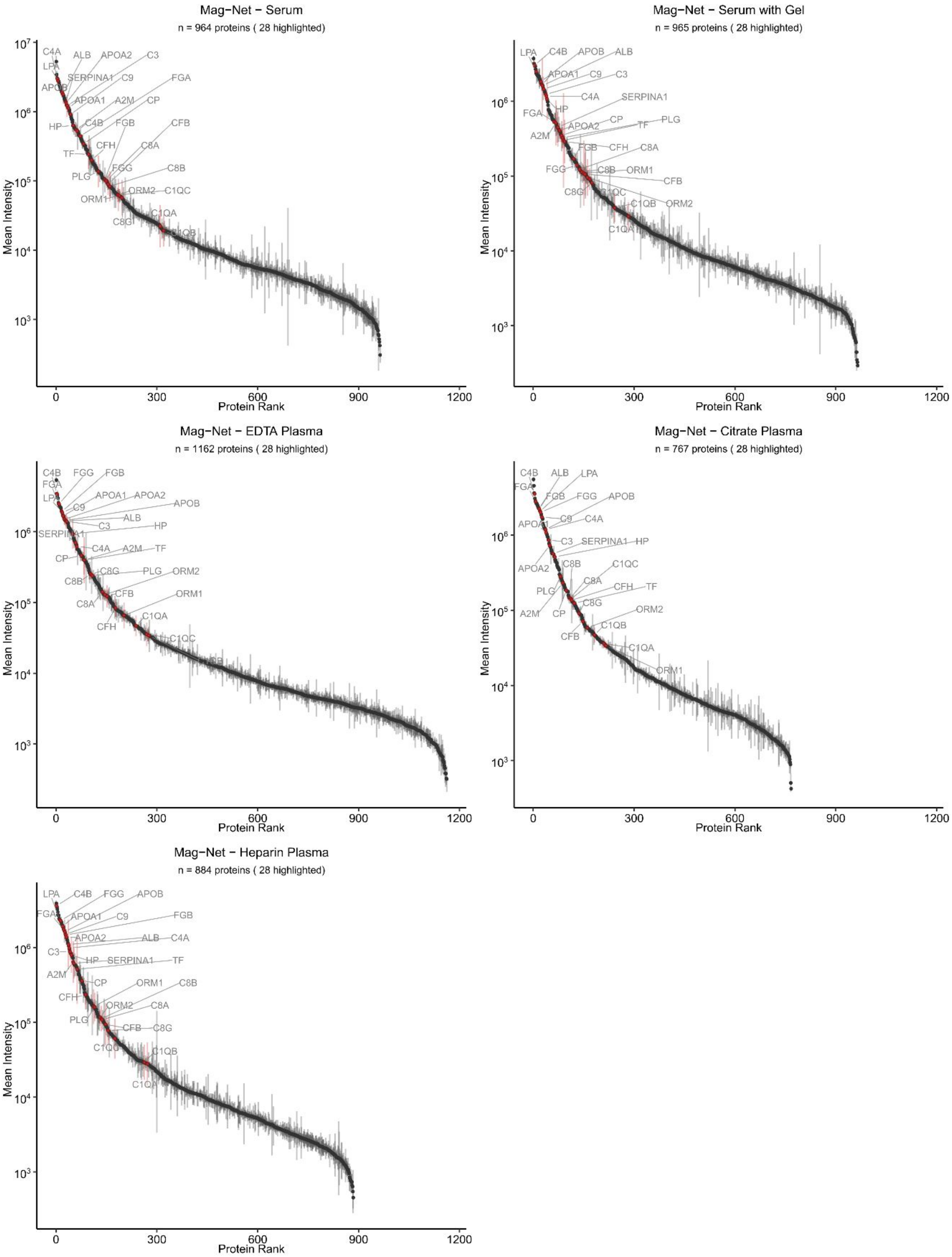

Supplementary Figure 9*. Rank plots of the combination of sample preparation method Mag-Net and all blood matrix types. Mean values from five replicates of protein group LFQ intensities are plotted in decreasing order. The 28 most abundant proteins are highlighted in red and labeled with their gene name.*

GO-term enrichment analysis for timsTOF HT data

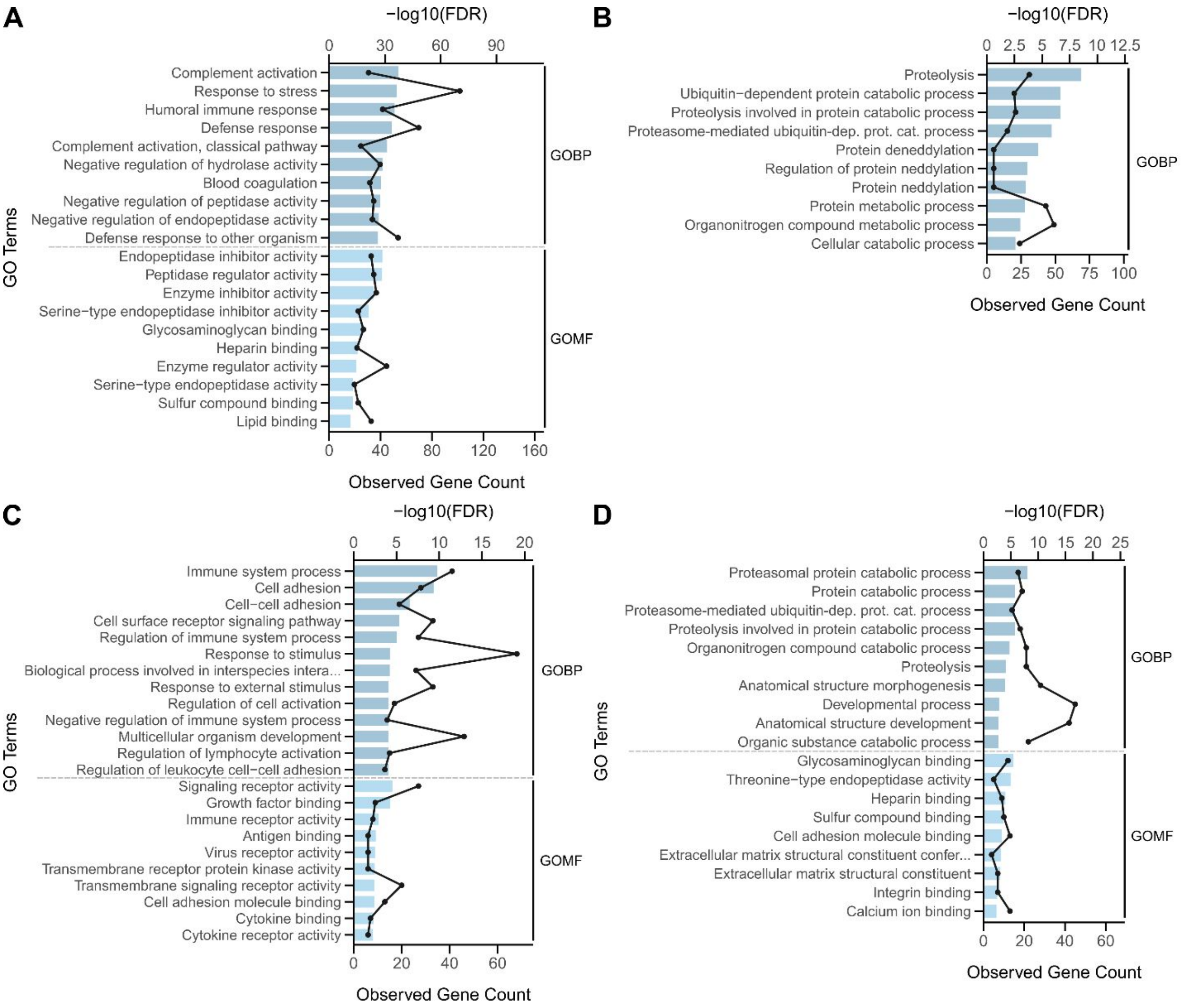

***Supplementary Figure 10**. Additional GO-term enrichment analyses of protein group clusters in data sets from timsTOF HT uniquely identified by: (A) every sample preparation method in each blood matrix type, (B) Mag-Net workflow with EDTA-plasma. (C) perCA-precipitation workflow with every blood matrix type and (D) Mag-Net workflow with every blood matrix type. Black lines indicate the observed gene count.*

Additional GO-term enrichment analysis for timsTOF HT data

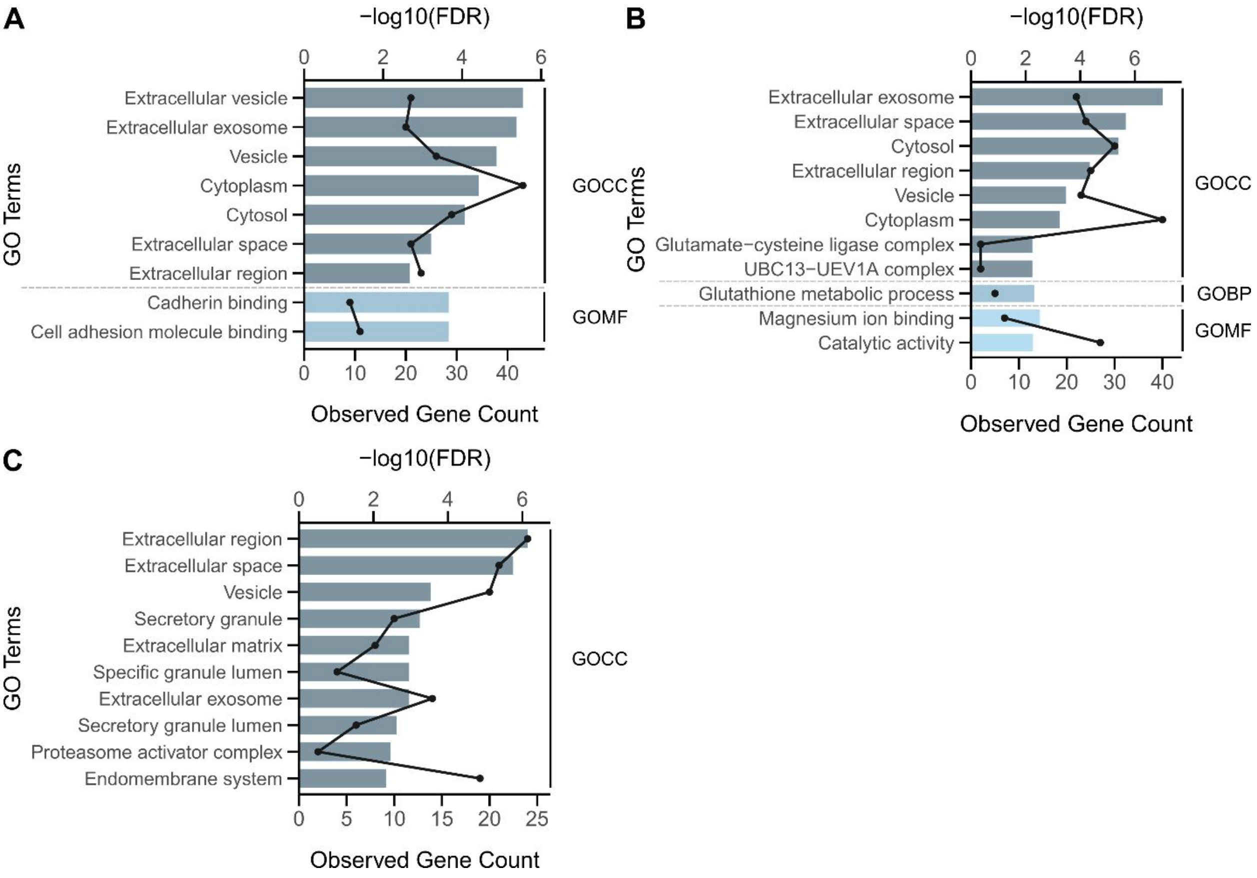

***Supplementary Figure 11**. Additional GO-term enrichment analyses of protein group clusters in data sets from timsTOF HT uniquely identified by: (A) ENRICH−iST workflow in heparin-plasma, (B) ENRICH−iST workflow in EDTA-plasma, (C) ENRICH−iST workflow in every blood matrix type.*

Upset plot and GO-term enrichment analysis of Q Exactive HF-X data

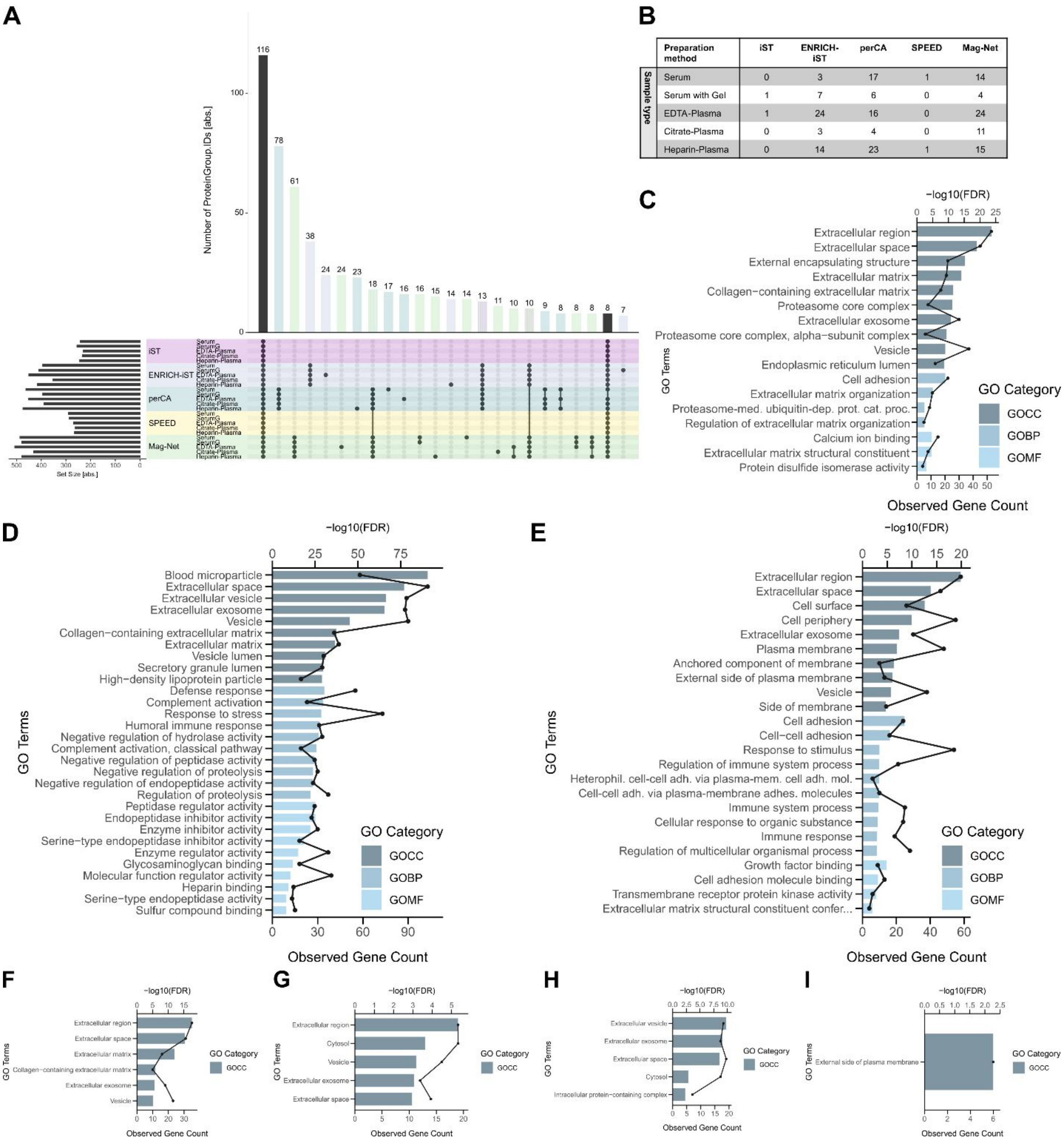

Preparation method	iST	ENRICH-iST	perCA	SPEED	Mag-Net
**Sample type**					
Serum	0	3	17	1	14
Serum with Gel	1	7	6	0	4
EDTA-Plasma	1	24	16	0	24
Citrate-Plasma	0	3	4	0	11
Heparin-Plasma	0	14	23	1	15

Supplementary Figure 12. *Upset plot representation of overlapping protein group identifications from measurements on Q Exactive HF-X. Visualization was set to only include cluster with more than 7 protein groups. (B) Tabular representation of individual clusters of protein groups that were identified by specific combinations of blood matrix and sample preparation method. (C-I) GO-term enrichment analysis of protein group clusters uniquely identified by: (C) Mag-Net workflow in each blood matrix type, (D) every sample preparation method in each blood matrix type, (E) perCA-precipitation workflow in every blood matrix type, (F) ENRICH−iST workflow in every blood matrix type, (G) Mag-Net workflow in EDTA-plasma, (H) ENRICH−iST in EDTA-plasma and (I) perCA in heparin-plasma.*

Comparison of log_2-transformed LFQ-intensities for timsTOF HT data

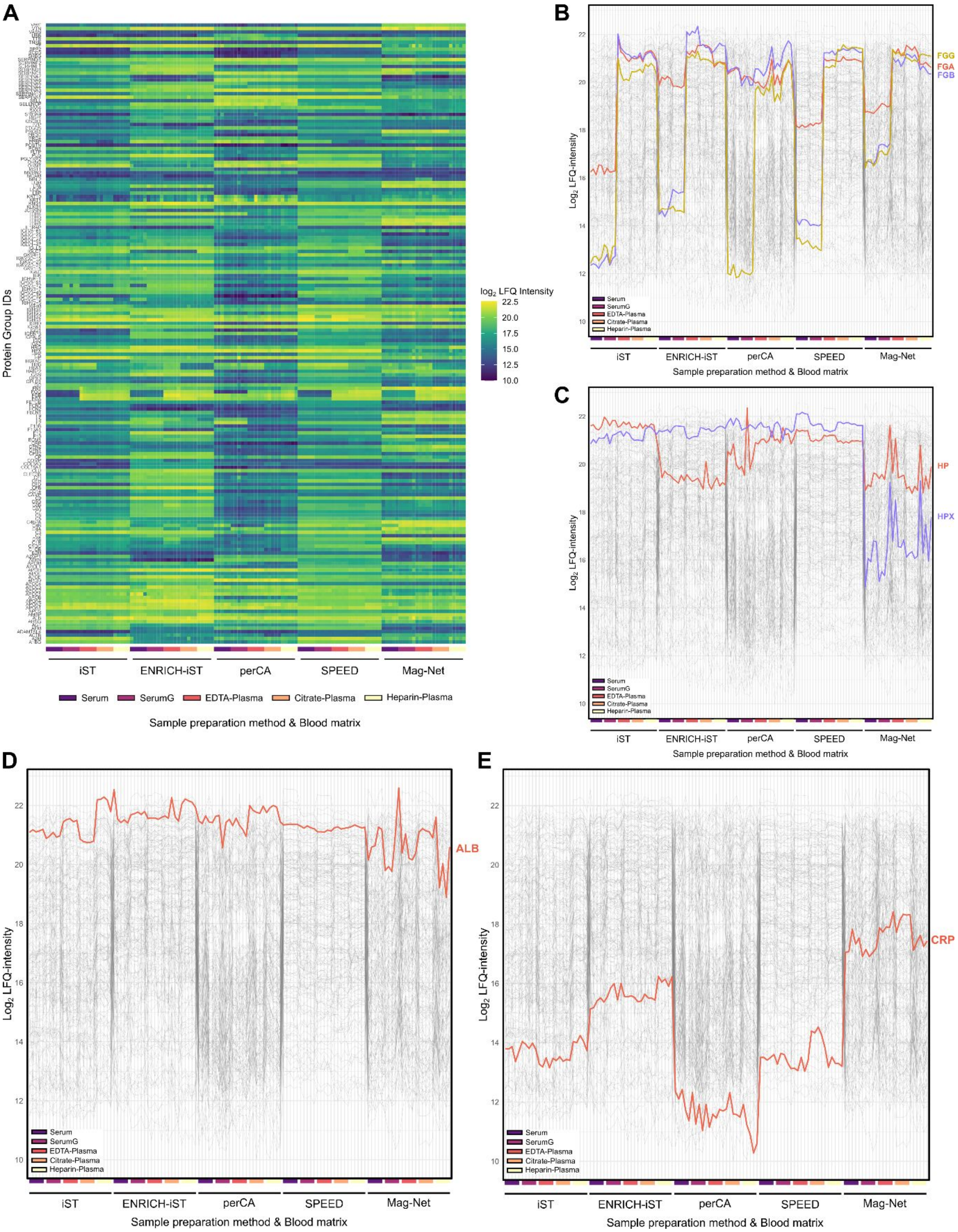

***Supplementary Figure 13**. Quantitative comparison of sample preparation methods and blood matrices on LFQ-level. (A) Heatmap representation of non-normalized, log_2-transformed LFQ-intensities from five sample preparation methods with five blood matrix types each. (B-E) Profile plots representing the different LFQ-intensities of clinically relevant protein groups.*

LFQ-intensities of known platelet markers for timsTOF HT data

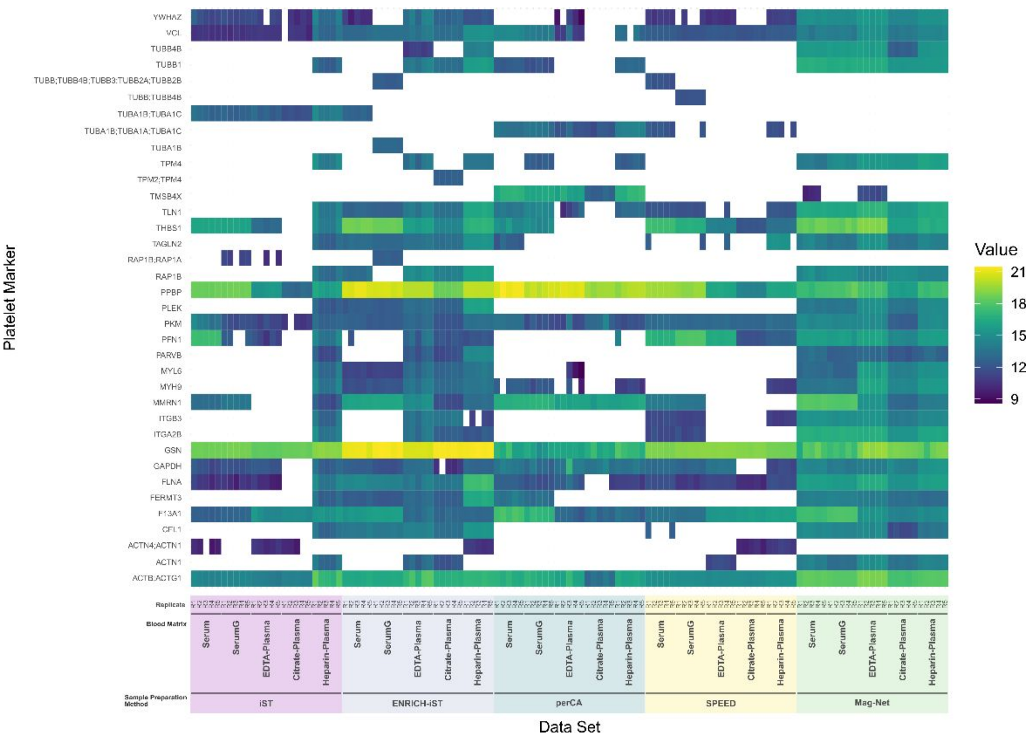

Supplementary Figure 14*. Non-normalized log$_2$-transformed LFQ-intensities of known platelet markers from measurements on timsTOF HT in heatmap representation.*

LFQ-intensities of known platelet markers for Q Exactive HF-X data

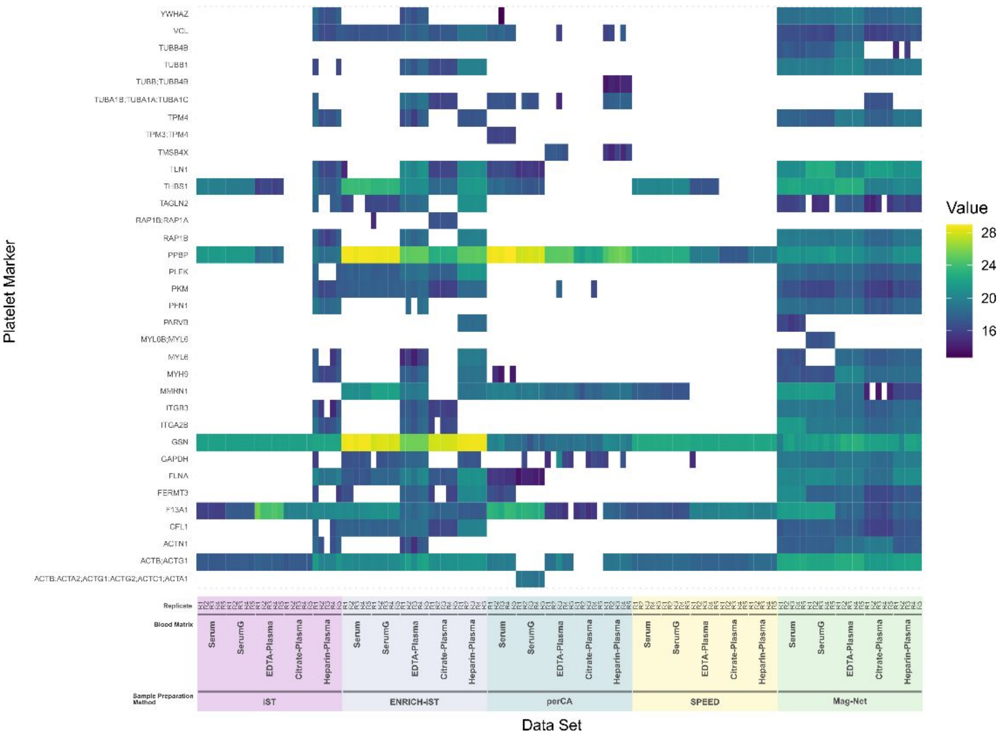

Supplementary Figure 15. *Non-normalized LFQ-intensities of known platelet markers from measurements on Q Exactive HF-X in heatmap representation.*
